# Polarization Upends Convention: Halogen Bonding Propensities of Main Group Halides

**DOI:** 10.1021/acs.jpca.4c06456

**Published:** 2025-01-15

**Authors:** Noah Robinson, Nam Pham, Kelling J. Donald

**Affiliations:** Department of Chemistry, Gottwald Center for the Sciences, University of Richmond, Richmond, Virginia 23173, United States

## Abstract

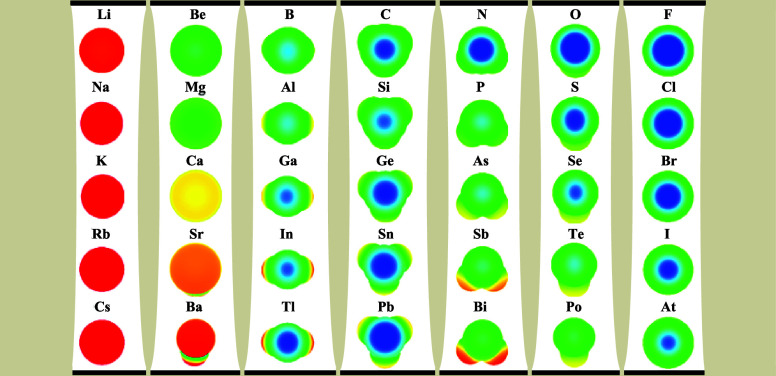

The propensities for sigma hole bonding by halogen atoms bonded to central atoms below period 2 in the periodic table remain to be systematically examined. Using iodine as our reference halogen atom, a comprehensive analysis of the tendencies for halogen and other forms of significant sigma hole bonding by simple compounds of main group atoms from H to At is accomplished. An examination of the structure and bonding of complexes formed by those iodine-substituted main group compounds and sigma donating bases (ammonia and trimethylamine) is performed to probe the viability of halogen bonding by heavy main group R_*n*_M–I compounds in particular, given the historic focus on period 2. We show that propensities for halogen bonding by F_*n*_M–I systems for certain columns of the main group vary anomalously as M gets heavier due to a polarization-induced escalation of the electrostatic potential on I. In certain cases, the positive potential at the sigma hole on I is weaker than that at sigma holes on the central M or geminal R atoms. Previously unexplored cases of strong halogen bonding by the fluoroiodides of heavy group 13 atoms are identified, and prospects for other sigma hole type interactions to polarized (main group) central atoms are elucidated.

## Introduction

The observation that a localized region of depleted electron density tends to emerge on a halogen atom, X, opposite the R–X bond if R is sufficiently electron withdrawing,^1^ greatly expanded our understanding of the halogen bonding phenomenon.^2−4^ That depletion of the electron density on X opposite the R–X bond arises from a shift of electron density from X into the R–X bonding region upon formation of the R–X bond,^1,2^ and the polarization of X can be so substantial that the area of depleted electron density (the so-called sigma hole) develops an exceptionally positive surface electrostatic potential.^3^ The term “halogen bonding”^5−8^—in analogy to hydrogen bonding^9−13^—refers to an interaction in which the polarized X atom (such as I in the F–I and F_3_C–I molecules, for example) establishes a weak bonding interaction with a base (e.g., NH_3_).

In halogen bonding, the region of positive potential that arises around the pole of the X atom opposite the covalent R–X bond (the general region indicated by the arrow here: R–I ←) aligns with the lone pair on the base, e.g., F–I···NH_3_, to give a roughly linear (∼180°) R–I···N bond angle. The extent and significance of any charge transfer^14,15^ from the base to the R–I antibonding orbital has been a topic of debate,^16−19^ but the existence of halogen bonding interactions as a class of weak interactions is not in question. Crystal structures featuring such interactions (see, e.g., refs (20−23)) had been identified long before they were christened “halogen bonds”.^20,24^ Yet, even now—and especially for extended systems—investigations of this bonding phenomenon remain rather scarce beyond a certain sliver of the periodic table, primarily halogenated organic compounds.

Investigations of halogen bonding (and other weak interactions that are linked to the sigma hole concept,^25^ such as so-called tetrel,^26^ pnictogen,^27^ and chalcogen bonding^28,29^) have progressed rapidly in organic and period 2 and 3 chemistry. Part of the reason for that is perhaps the important roles of nitrogen, oxygen, the halogens, phosphorus, and sulfur containing compounds in (hetero)organic chemistry and drug design and the common occurrence of N and O centers as donor sites (lone pair sources) in popular bases.^30^ There is also the ever-growing body of evidence for the presence and consequences of various types of weak sigma hole interactions in organic chemistry and biochemistry^31−33^ (and the attending interests in identifying new occurrences and even novel pharmaceutical and other applications of halogen bonding).^34^ Here, we are thinking, for example, about cases in which halogen bonding and other sigma hole interactions strongly influence if not control structural preferences in organic crystal structures^35−37^ and an apparent role of such interactions in the conversion of thyroxine (T4) to triiodothyronine (T3).^31,38,39^ On the computational side, even relatively large organic molecules are rather inexpensive to study at high levels of theory compared to systems with many heavy atoms.

Yet, complexity is matched by both intrigue and rich intellectual rewards as we go down the periodic table, even if we consider the main group atoms alone. Period 2 is a vibrant narrow strip of the periodic table, but a vast universe lies beneath it. Another reason that period 2 compounds have been popular for studying halogen bonding in particular is the high electronegativity of the elements in period 2 compared to their heavier group congeners.

The halogen atom, X, in the CH_3_X molecule is sure to have a more positive sigma hole than that on SiH_3_X, for instance, since C is significantly more electronegative than Si or, more precisely, since the H_3_C– group or fragment is more electronegative than the H_3_Si– group. So any halogen bond formed between CH_3_I and a given base, CH_3_–I···Base, is expected to be stronger than the analogous “SiH_3_–I···Base” complex. But, as we have pointed out in a previous report,^40^ electronegativity varies erratically going down group 14 on both the Pauling and Mulliken (valence state) scales,^41^ with Pb having the second largest electronegativity after carbon. So, going from C to Pb down group 14, the trend in the electronegativity of the central atom, M, predicts neither a monotonous decrease in the group electronegativity of any R_3_M– fragment nor a continuous decline in the magnitude of the sigma hole potential on I in the corresponding MR_3_I molecule as M gets larger.

As it turns out, the sigma hole on I in the group 14 MH_3_I molecules actually does shrink continuously as M gets larger and heavier from C to Pb. But that is not the case at all for the MF_3_I systems.^40,42^ The extreme polarization of M by the F substituents in the MF_3_I molecules leads inductively to an increasingly rapid spatial expansion and strengthening of the sigma hole on I after M = Si. Indeed, PbF_3_I has the largest sigma hole on I among the group 14 MF_3_I molecules, and the strongest group 14 MF_3_I···NH_3_ halogen bond complex is not CF_3_I···NH_3_ but PbF_3_I···NH_3_!

Hitherto, however, a comprehensive examination of the nature of halogen bonding by a full slate of main group halides has been missing from the literature. The likelihood of anomalies similar to those of the group 14 case arising elsewhere in the main group (where halides of heavier atoms in a given group form stronger halogen bonds than their period 2 counterparts) remains to be investigated. Studies of tetrel, pnictogen, and chalcogen bonding have been undertaken, in some cases for a number of atoms below period 2 in groups 14, 15, and 16, respectively, but a more systematic assessment is warranted on those fronts as well. We undertake here, therefore, a detailed analysis of the halogen bonding abilities of main group compounds with the general formula R_*n*_M–I (for R = H and X), and we consider in that context too the nature of sigma holes induced elsewhere on those molecules—on the central M and geminal R atoms.

We report certain previously unidentified patterns in the maximum electrostatic (isodensity surface) potentials,^43^*V*_s,max_, at the sigma hole on I in the MF_*n*_I compounds of main group atoms. The presence of even one lone pair on the central M atom can substantially alter, we find, the impact of fluorination on trends in halogen bonding by I in neutral MR_*n*_I systems. Of note, the erratic trend in *V*_s,max_ that was thought previously to be isolated to group 14 is shown to arise in group 13 as well! Applications of halogen bonding in crystal engineering and other areas are growing. Additional insights into the capacity for sigma hole bonding by compounds of heavy atoms are vital for identifying and capitalizing on new, interesting, and useful applications in inorganic structural chemistry, materials science, and crystal engineering.

## Methods

The geometrical data and all of the additional computational results presented in this work have been obtained using the density functional ωB97XD method^44^ as implemented in the Gaussian 16 suite of programs.^45^ The cc-pVTZ basis sets^46,47^ have been employed for all elements considered in this work that are lighter than Kr in the periodic table, except for K for which the cc-pVTZ basis set is unavailable in the Gaussian 16 software. For that element, and for the elements beyond Kr, we employed the Stuttgart–Cologne small-core energy-consistent relativistic (multiconfigurational Dirac–Hartree–Fock) MDF pseudopotentials (without the spin–orbit parts, which were unavailable for our calculations) and the corresponding (MDF) triple-ζ quality basis sets.^48,49^ The electrostatic potentials (ESPs) on the 0.001 au isodensity surfaces of the species reported in this work have been generated using the GaussView 6 software^50^ and the Chemcraft program^51^ was used in collecting geometrical data for optimized structures. The maximum potentials on the terminal and central atoms under consideration were determined using the MultiWFN program^52,53^ by determining and ranking the local minima and maxima across the potential energy surface of each molecule. The interaction energies associated with halogen bond formation between main group iodides and selected bases are assessed in tandem with the basis set superposition error (BSSE) estimated using the counterpoise correction^54,55^ as implemented in the G16 suite of programs. The corresponding enthalpy and free energy changes have been calculated as well by subtracting the sum of the relevant (enthalpy or free energy) values for the isolated molecules from that of the halogen bonded complex, with subsequent inclusion of the BSSE correction.

## Results and Discussion

**Figure 1:**
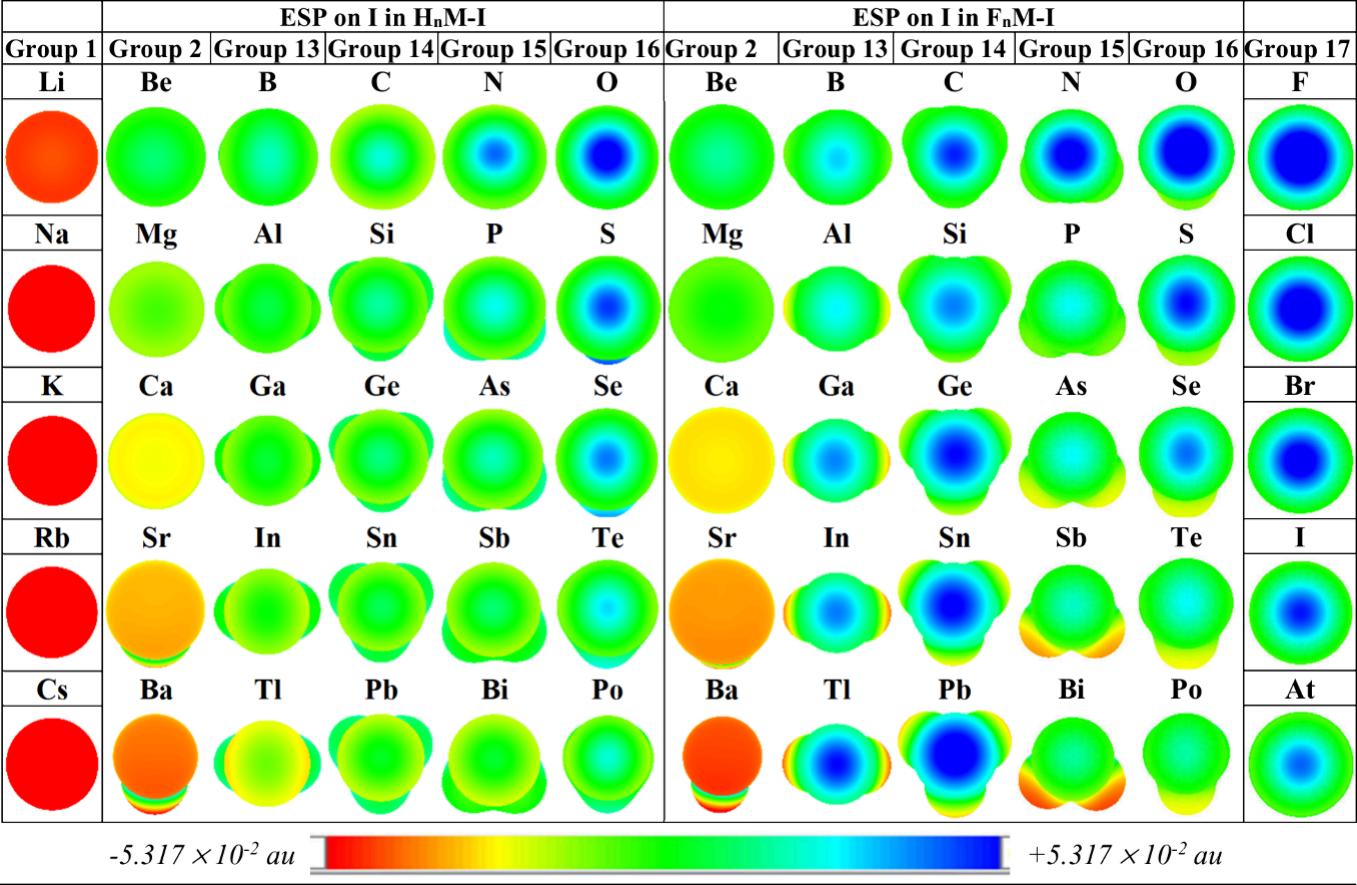
Electrostatic potentials (ESPs) mapped (in the range ±5.317 × 10^–2^ au) onto the 0.001 au isodensity surface for R_*n*_M–I main group monoiodides. M is the central atom indicated above each map, and R = H (left) and F (right). NOTE: Groups 1 and 17 are diatomic (M–I) with no R group.

The computed surface electrostatic potential (*V*_s_) maps for main group monoiodides saturated by terminal H or F atoms are shown in Figure 1. We exclude group 18, as well as the main group elements beyond At (which are less abundant and unstable), from consideration. For all of the elements in a given group, we consider only the valence that is characteristic of the period 2 element (i.e., one for groups 1 and 17, two for groups 2 and 16, three for groups 13 and 15, and four for group 14).

The ESP maps in Figure 1 are oriented such that the iodine atom in the R_*n*_M–I molecule is facing the reader, pointing directly out of the plane of the page, with the rest of the molecule (R_*n*_M−) immediately behind and (partially or fully) obscured by the iodine atom. In this section, R = H and F. The group 1 and 17 systems are monovalent (M–I; i.e., *n* = 0), so the identity of R is irrelevant in those two cases, and only one column is needed in Figure 1 for those two groups.

Instead of repeating those two columns in both the R = H and R = F sections in Figure 1, therefore, we have placed the group 1 maps at the front in Figure 1 (preceding the R = H section) and the group 17 maps at the very end (after the R = F section)—see Figure 1. That arrangement, we think, is still conducive to discerning trends in the ESP maps. The data for the H–I molecule (i.e., for M = H, which is not in Figure 1) is shown in Figure S1 in the Supporting Information (SI). The largely unreactive group 18 elements—for which R_*n*_M–I species remain unknown—are ignored, but, of note, strong sigma holes are induced on Kr and Xe in their (oxy)fluorides.^25,56^

The selection of R = H and F as the saturating substituents of the main group monoiodides that we consider here allows us to assess the impact of changes in the electron withdrawing power of geminal substituents on the nature of the sigma hole on a reference I atom that is common to the R_*n*_M–I molecules discussed. The electrostatic potential maps shown in Figure 1 are qualitative proxies for the propensity for halogen bonding by the I atom bonded to the relevant R_*n*_M– fragment. As we mentioned in the Introduction, the influence of geminal R substituents has been examined previously for iodides of certain period 2 and group 14 central atoms.^40,57^ The current study has allowed us to analyze in an unprecedented way the capacity for halogen bonding by halides of atoms from across the entire main group and to quantify the influence of two types of R substituents with very different electron withdrawing abilities on that halogen bonding capacity.

The electrostatic potentials of the hydrides and halides of the main group elements shown in Figure 1 tell an intriguing story. For the hydrides (on the left-hand side of Figure 1), the positive potentials on the iodine center, evident as a blue region on the center in the individual ESP maps in Figure 1, are lost or become increasingly weak (the maps becoming greener, tending toward yellow and red) going down each group. That monotonous decline in the positive potential on I is observed for the group 17 species (on the far right) as well, and although it is difficult to see, since the potential on I in Li–I is already negative (orange-red), the potentials also get less positive (more negative) going down that group too through to Cs–I. So, the hydrides (and the group 1 and 17 iodides) follow precisely the behavior that a brilliant undergraduate might predict based on general expectations of how the electronegativity of atoms (χ(M)) changes going down and across the main group: if the M atoms become less electronegative going down each group, it is unsurprising that they increasingly fail to polarize the I atom enough to induce a positive sigma hole (or any sigma hole at all) on the potential surface.

But the fluorides exhibit a very different kind of behavior. When the hydrogen substituents are replaced with fluorines, the trends observed for the hydrides disappear. Sure, the sigma holes on I in the fluorides are magnified relative to their hydride analogues, but the relative strengths of the sigma holes (evident in the intensities of the blue regions on I in Figure 1) are completely reordered for both groups 13 and 14 (see the R = F section of Figure 1). The ESP images arrayed in Figure 1 reproduce fully, therefore, the anomaly identified previously for group 14 compounds^40,57^ but expose, as well, an identical situation elsewhere (in group 13) in the periodic table. For the group 13 hydrides (see the left half of Figure 1), positive sigma holes (blue regions) are not easily discerned even for B (the most electronegative atom in that group), and they become increasingly weak as we go down that group. But the situation is very different for the fluorides. In that case (for group 13 on the R = F side of Figure 1), the order is almost completely reversed; the strongest sigma hole is observed (based on the ESP maps alone) for thallium! Indeed, in all of row 6, only F_3_Pb–I and At–I appear to have stronger or comparable sigma holes on the iodine center.

Curiously, deviations from the “conventional” trend seen in the hydrides are observed, among the fluorides, only in groups 13 and 14. Compared to the corresponding hydrides, the group 2, 15, and 16 fluorides have more massive and more positive sigma holes on the I center (Figure 1), but the size and strength of the iodine sigma holes decrease monotonously for both the hydrides and the fluorides as M gets larger for all three of those groups (Figure 1).

**Figure 2:**
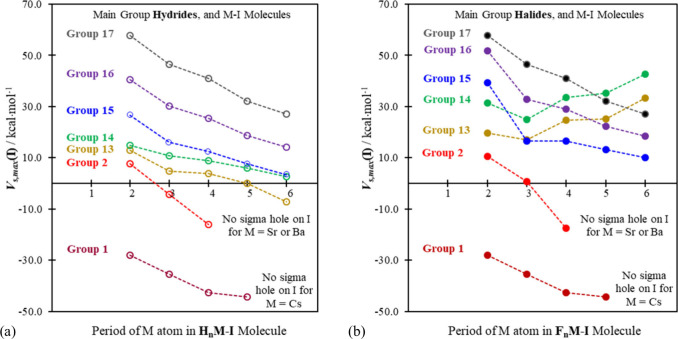
Plots of *V*_s,max_/kcal·mol^–1^ at the sigma hole on I in R_*n*_M–I molecules for (a) R = H and (b) R = F, vs the period of M. Here *n* = 0, 1, 2, 3, 2, 1, and 0 for groups 1, 2, and 13–17, respectively. For reference, the R-independent group 1 and 17 cases (M–I; *n* = 0) are included in both graphs.

The maps in Figure 1 reflect actual ESP values on the 0.001 au isodensity surfaces, but our analysis has been rather qualitative so far. For a more rigorous assessment, therefore, of the influence of fluorination on the strengths of the sigma holes, we computed the values of the maximum potentials within the iodine sigma holes (*V*_s,max_(I)) for all of the species considered, and we plotted those data separately for the hydrides and the halides in Figure 2.

We will discuss the key trends in Figure 2 presently, but a few intriguing, if peripheral, features of the data should be highlighted at the outset. We find that (for both R = H and R = F) there is no sigma hole (not even a negative maximum) on I in CsI, SrRI, or BaRI. Instead, there is a negative ESP minimum (*V*_s,min_) on I in those molecules. This observation is not too surprising; since Cs is so electropositive (the least electronegative of the M atoms considered in this work), we expect I to pull electron density away from Cs, not vice versa. And, in addition to the low electronegativities of the Sr and Ba atoms, SrHI, SrFI, BaHI, and BaFI are bent molecules^58,59^—instances of the well-established bending phenomenon in compounds of heavy group 2 atoms.^60−62^ That reduction in the symmetry (and the associated rehybridization going from linear to bent) such that the R and I substituents are not aligned, combined with the softness of Sr and Ba, apparently succeeds in limiting any inductive influence of the F (or H) substituents on the geminal I site in those Sr and Ba molecules. A sigma hole is identified on the I center in all of the other systems considered, even if the *V*_s,max_(I) values are negative for almost all of the group 1 and 2 molecules (for the M atoms above Cs and Sr) where sigma holes exist (see Figure 2 and Table S1).

More to the central question of this work, the computed *V*_s,max_(I) values for the sigma holes on I in the R_*n*_M–I molecules (see Figure 2) confirm our expectations based on the ESP maps in Figure 1 that (for a given M) the F_3_M– fragment generally induces a much more positive sigma hole on I compared to the H_3_M– analogue. We included the R-independent (groups 1 and 17) data on both the hydride (Figure 2a) and the fluoride (Figure 2b) graphs, so it is easy to see from a comparison of the two panels in Figure 2 that the *V*_s,max_(I) values shift up and away from the group 1 line and toward the group 17 line going from R = H to R = F. And, the most prominent feature in that regard is a disproportionate escalation in the magnitude of the *V*_s,max_(I) values for group 13 and 14 atoms after period 3 in Figure 2b, which reflects in remarkable detail the pattern betrayed already by the ESP maps in Figure 1. In Figure 2a, *V*_s,max_(I) decreases monotonously as we go from left to right (i.e., down each group; cf. Table S1) in line with the qualitative trends for hydrides in Figure 1. In Figure 2b (where, recall, the group 1 and 17 data are identical to those in Figure 2a), the other five series of *V*_s,max_(I) values (for R = F) decrease monotonously only for groups 2, 15, and 16.

For groups 13 and 14, the data are completely in line with the message of the dramatic ESP maps in Figure 1. The *V*_s,max_(I) values decrease going from period 2 to period 3 (from B to Al and from C to Si) but escalate thereafter, such that F_3_Al– and F_3_Si– induce the smallest (least positive) *V*_s,max_ values on I in groups 13 and 14, respectively, for the fluorides and the largest (most positive) *V*_s,max_(I) values are obtained for M = In and Tl in group 13 and M = Sn and Pb in group 14 (see Table S1). In Figure 2b, this disruption of the monotonous trends observed for the other groups is obvious as the brown and green check mark (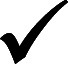) shaped lines rise rapidly toward and above the lines for groups 16 and 17 after period 3. This result predicts that two of the most positive sigma holes possible for I on any main group central atom in the periodic table are those (yet to be probed experimentally) on I in the F_3_Pb–I and F_2_Tl–I molecules. The computed *V*_s,max_(I) values obtained for those two cases are comparable to and even exceed that for F_3_C–I, which has received significant attention over the years as an excellent model system for halogen bonding.

We have not been able to locate any earlier study probing and contrasting the halogen bonding tendencies of the heavy group 13 compounds. The toxicity of some of the heavier main group elements, such as Tl and Pb, imposes a limitation on their use in several (especially biologically relevant) applications. Moreover, the inert pair effect and the resulting preference for lower oxidations states, such as +1 and +2 states for Tl and Pb, respectively, means that their tri- and tetra-valent species (TlR_2_I and PbR_3_I) may be of interest in some areas of study (including surface and materials chemistry) but not in others. Yet, bottleable thallium(III) and lead(IV) halides are known—thallium trifluoride and lead tetrafluoride are stable solids under ambient conditions with established crystal structures and melting points above 500 °C.^63−65^ We have found no experimental investigation of the properties or complexes of the monoiodofluorides of these two compounds, but our results (Figure 2b) show that they have substantial *V*_s,max_(I) values and are expected to form, for a given base, halogen bonds comparable to and even stronger than those of species such as CF_3_I, BrI, and I_2_ that have similar *V*_s,max_(I) values in Figure 2b. Lighter mixed halides (BF_2_I,^66^ CF_3_I,^67^ and SiF_3_I^68^) have been prepared by redistribution (processes of the general type MF_*x*_ + M′I_*x*′_ → MF_*x–y*_I_*y*_ + M′I_*x*′*–y*_F_*y*_),^66−68^ and we consider that such redistribution reactions may be viable for generating heavier group 13 and 14 MF_*n*_I species as well.

A few other exceptional features of the data in Figure 2 remain to be highlighted. The group 17 X–I compounds have the strongest *V*_s,max_(I) values in their respective rows, except in rows 5 and 6 where they are surpassed by the group 13 and 14 systems. Indeed, the diatomic F–I and Cl–I and triatomic FO–I molecules are all predicted to have the largest *V*_s,max_(I) values and thus the strongest halogen bonding interactions of all main group halides. In groups 15 and 16, NF_2_I and OFI generate particularly strong *V*_s,max_(I) values relative to their heavier congeners and are expected, thus, to form the strongest F_*n*_MI···Base complexes in their respective groups. For NF_2_I and OFI, the central atoms themselves (N and O) are already quite electronegative, and the considerable N ← I and O ← I polarization is enhanced by the inductive effect(s) of the F substituent(s). Remarkably, NF_2_I is the only group 15 fluoride with *V*_s,max_(I) greater than that of its group 14 counterpart (Figure 2b and Table S1), and we will see shortly that a convergence of the atomic electronegativities of group 14 and 15 atoms as we go down the periodic table can help us to rationalize that observation.

Our results offer, we hope, some impetus for the experimental investigation of the sigma hole bonding ability of novel halides (such as heavy main group fluoroiodides and the ternary (MF_*n*_I) derivatives of NF_3_,^69−71^ or OF_2_, though the latter is much less stable than NF_3_^72^) beyond the more commonly studied halides of the lighter group 14 elements.

### Evidence from Structure and Energetics

To assess the practical implications of the anomalous patterns in *V*_s,max_ at I in group 13 and 14 fluoroiodides for halogen bonding, we optimized the geometries and determined the interaction energies, enthalpies, and free energies for group 13 and 14 R_*n*_M–I···NR′_3_ complexes (for R = H and F and R′ = H and CH_3_). And, for comparison, we obtained the corresponding data for the group 17 M–I···NR′_3_ systems as well. The monotonous decrease in the *V*_s,max_ value at I in the group 17 iodides (Figure 2) as M gets heavier is unambiguous, so the group 17 systems serve as a simple reference set against which to compare the computed ESP data discussed so far and the structure and thermodynamic stability of the group 13 and 14 halogen bonded complexes. Moreover, the group 17 systems exhibit, for most of the rows in the main group, the largest *V*_s,max_(I) values (Figure 2) with convergence occurring only due to the polarization-induced escalation of *V*_s,max_(I) for the heaviest group 13 and 14 fluoroiodides.

**Figure 3:**
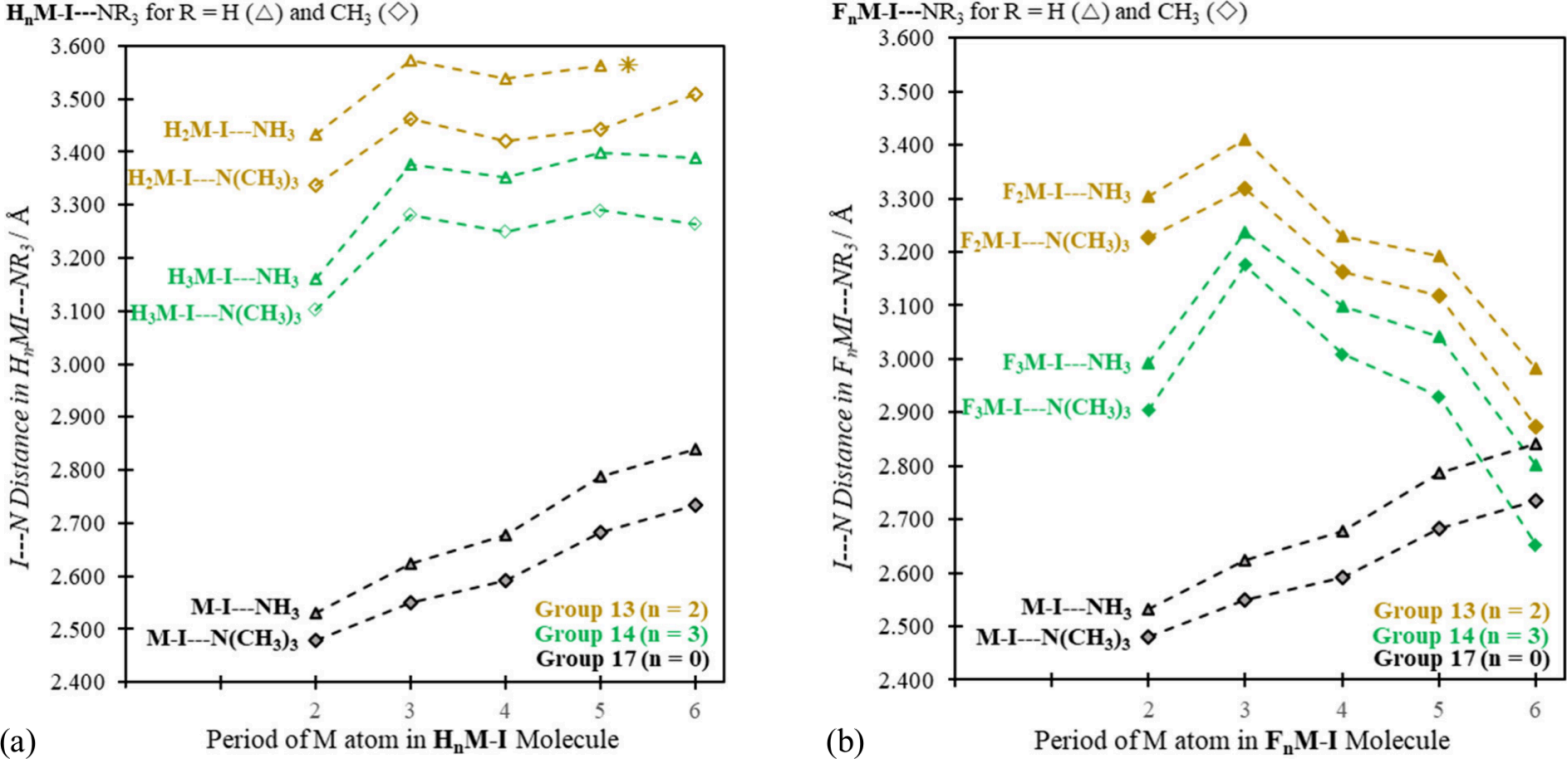
Optimized I···N distances in Å units in minimum energy R_*n*_M–I···NR′_3_ complexes for group 13, 14, and 17 M atoms, for R = (a) H and (b) F, with R′ = H and CH_3_. (No H_2_Tl–I···NH_3_ sigma hole complex was obtained.)

**Figure 4:**
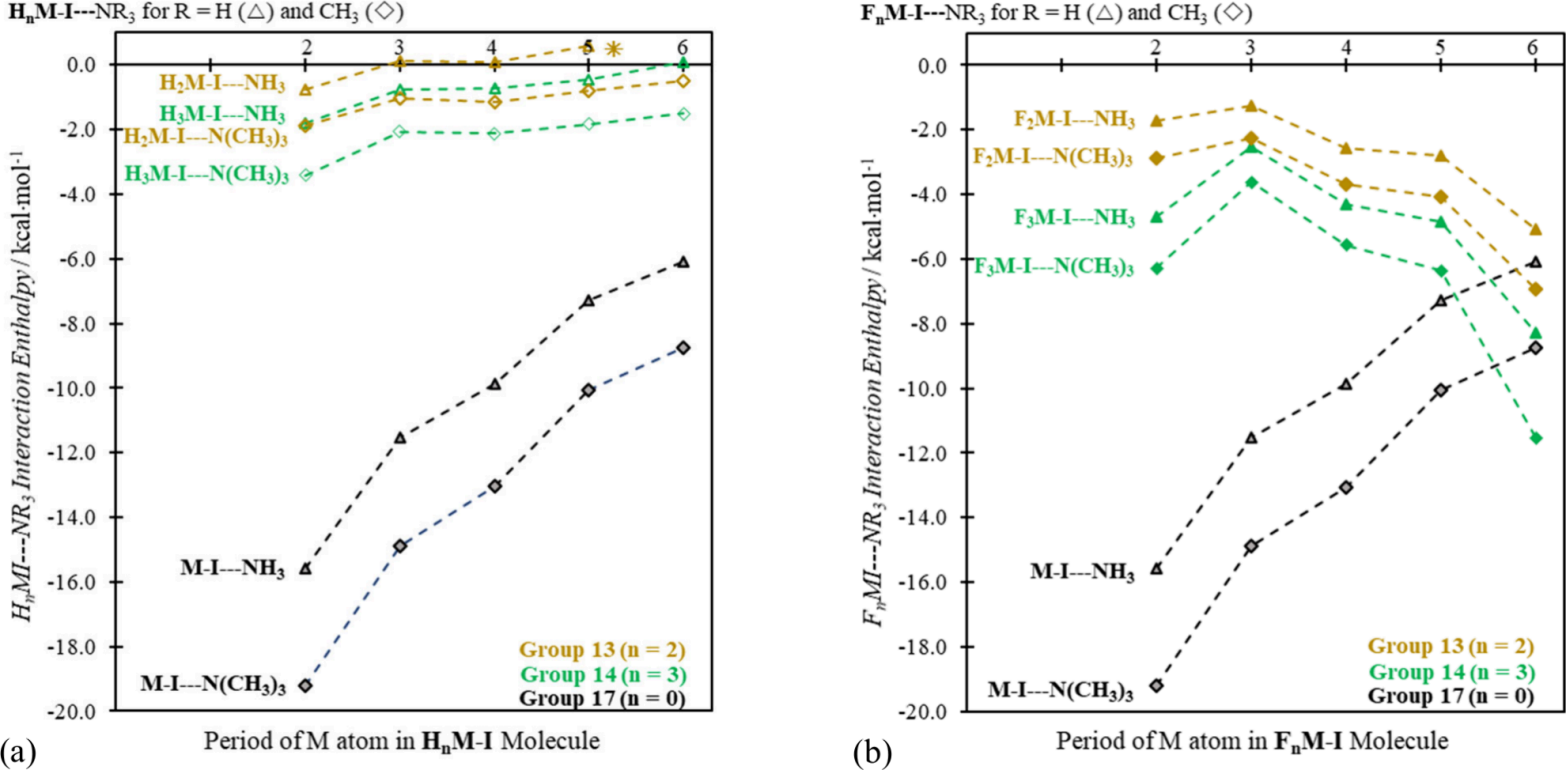
BSSE corrected interaction enthalpies (Δ*H*^BSSE^) in kcal·mol^–1^ units for group 13, 14, and 17 R_*n*_M–I···NR′_3_ systems, for R = (a) H and (b) F and for R′ = H and CH_3_. (No H_2_Tl–I···NH_3_ sigma hole complex was obtained.)

Based, therefore, on the results in Figure 2, one expects that the group 17 M–I···NR′_3_ complexes will (i) have consistently shorter and stronger I···N halogen bonds than those of the group 13 and 14 hydrides (H_*n*_M–I···NR′_3_) but (ii) for the fluorides, the length and strengths of the I···N bonds of the group 13 and 14 complexes will converge with those of the corresponding group 17 systems as M gets heavier. And that—as we show in Figure 3 (for I···N separations) and Figure 4 (for interaction enthalpies)—is precisely what we observe.

The H_2_Tl– fragment induces a sigma hole on I in H_2_Tl–I, but that *V*_s,max_(I) value (the most positive (least negative) point in the center of the sigma hole) is negative (see Figure 2). That situation naturally favors I···Base repulsion, and no H_2_Tl–I···Base halogen bond complex was located computationally. For all of the other group 13, 14, and 17 systems, computed Cartesian coordinates, from which specific geometrical parameters can be obtained, are available as .xyz files in the SI. In addition to the enthalpies in Figure 4, The BSSE corrected energies and free energies are also provided in the SI (Figures S2–S4). For ease of comparison, the same scale is used for plots “(a)” and “(b)” in Figures 3 and 4, and the differences between the hydrides and the fluorides (going from “(a)” to “(b)” in both figures) are striking. They reflect unequivocally the distinctions observed in Figure 2 for the range of *V*_s,max_(I) values for the isolated hydride and fluoride molecules. In Figures 3b and 4b the I···N distances and enthalpies, respectively, for the heaviest group 13 and 14 complexes fall squarely within the range of the corresponding values for the group 17 species. And the check mark pattern (now inverted, since high *V*_s,max_(I) values in Figure 2 lead to shorter bond distances (Figure 3) and more negative (lower) interaction enthalpies (Figure 4)) is well reproduced in Figures 3b and 4b.

These results provide rigorous confirmation, therefore, that the escalation of *V*_s,max_(I) by the inductive polarization of iodine by F substituents, which is observed in both groups 13 and 14 (Figure 2), is expected to have direct and qualitatively predictable consequences for the structure and bonding of any halogen bonded pair that highly polarized heavy group 13 and 14 iodides may form. We show only the enthalpy data in the main text, but the same trends are obtained for *ΔE* and *ΔG* (see Figures S2–S4 and the actual values in Tables S2–S4), except that, for any given group of compounds, the *ΔG* values are noticeably more positive (shifted higher up along the *y*-axes of the graphs) due to positive entropic contributions to the interaction free energy. The thermodynamic stability of these complexes, as is typical of weak gas-phase interactions, is expected to be particularly sensitive to the temperature.

### Modeling Sigma Hole Variations across the Main Group

Having surveyed *V*_s,max_(I), as reported in Figures 1 and 2 for the whole main group of the periodic table, it remains now to account comprehensively for the emergence, in the middle of the table, of the unusual variations in *V*_s,max_(I) for fluoroiodides—with its entailments for the relative strengths of halogen bonds. The anomaly, which was identified in isolation for group 14,^40,57^ is found to arise for group 13 systems as well.

**Figure 5:**
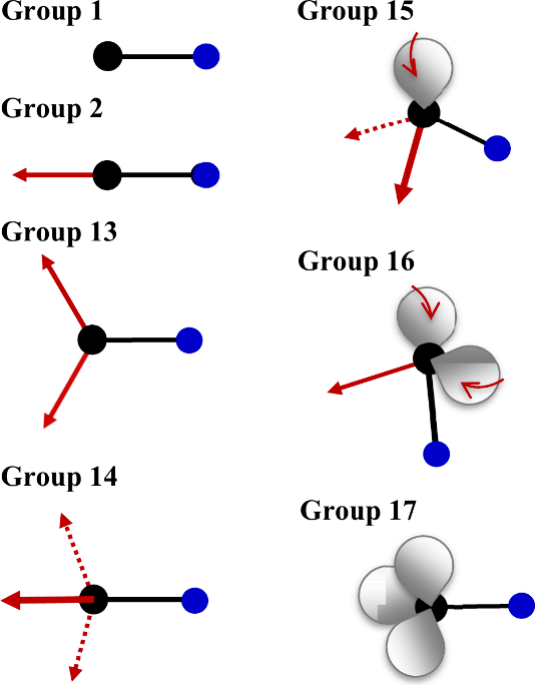
Illustration of the net flow of electron density relative to the M center (black circle) in main group MF_*n*_I molecules (away from M toward F (straight red arrows) and the resulting enhanced localization (curved arrows) of the lone pair(s) (gray lobes) on M). The I site is in blue. For groups 1 and 17, *n* = 0; there is no F substituent.

We employ in Figure 5 a simple picture to illustrate the flow of electron density in M–F bonds (straight arrows) and the contraction or localization of the lone pair(s) on M (curved arrows). Going from group 1 to group 17, the number of R substituents increases from *n* = 0 at group 1 to *n* = 3 at group 14 and decreases thereafter to *n* = 0 at group 17. Based on electronegativities alone, the polarization of M by F will be much more substantial than any polarization of M by H (or by most other substituents), so a general increase in *V*_s,max_(I) going from R = H to R = F, with substantial jumps in *V*_s,max_(I) at groups 13 and 14 (as *n* goes to 2 and 3, respectively), is understandable.

Yet, why does the inductive response by I to fluorine substitution peak at the heaviest M atoms in groups 13 and 14 only? For the group 1 and 17 iodides (as depicted in Figure 5), there is no geminal M–F bond. For the group 2 systems, there is one substituent immediately opposite the M–I bond (for the linear Be, Mg, and Ca systems at least), but the group 2 atoms themselves are sufficiently soft and electropositive (and increasingly so going down the group) that the fluoride *V*_s,max_(I) values show the same trend in sigma hole strengths as the hydrides as M gets heavier: For both R = H and R = F, *V*_s,max_(I) decreases as M gets larger—and disappears, as noted above, for M = Sr and Ba. At group 13, the electronegativity of M itself increases slightly (relative to the preceding group 2 atom), and the number of polarizing F substituents increases by one as well. And both factors favor enhancement of the through-bond inductive polarization of the I center. As indicated in Figure 5, the net effect of those changes going from group 2 to group 13 is a significant flow of electron density away from the M center and inductively, thus, from I into the M–I bonding region to generate larger *V*_s,max_(I) values compared to those achieved by the neighboring group 2 species. And all of those effects are amplified even more in group 14. The group 14 atoms are inherently more electronegative than their group 13 neighbors, and they have three F–M bonds, which unite to magnify the polarization of the I center—expanding and strengthening the sigma hole—relative to even the group 13 case (Figure 2b).

Going from group 14 to group 15, the Pauling electronegativity increases somewhat (for the lighter elements at least; see Table S5), so, compared to their group 14 neighbors, the group 15 M atoms might be expected to induce stronger sigma holes on I in their F_*n*_M–I molecules. But two factors work to counter the inductive influence of the geminal F substituents on I. Namely, (i) a reduction in the number of F substituents from three to two going from group 14 to group 15 and (ii) the replacement of that M–F bond with a lone pair on M, which responds to M → F polarization by localizing more significantly (becoming less diffuse) on the M center, thus blunting the influence of any M → F polarization on the M–I bond (see Figure 5). At group 16, the electronegativity of M increases even more, but the removal of another M–F bond and the presence of a second lone pair on M diminish the net inductive influence on I. For F–O–I, the influence of the electronegative atom by itself (reinforced by the presence of the electron withdrawing F substituent) on the O–I bond is significant as we mentioned above, but the *V*_s,max_(I) values fall off rapidly for the group 16 systems after oxygen (Figure 2b). At group 17, the atoms are generally electronegative enough by themselves to induce the largest *V*_s,max_(I) for each row in the main group, except at periods 5 and 6, where that preeminence is eventually lost to the group 13 and 14 fluorides (Figure 2b).

The grand escalation of the iodine polarization in the heavy group 13 and 14 fluoroiodides emerges, thus, as a result of the inherent polarizability of the central M atoms themselves, coupled with the absence of lone pairs, and the presence of a higher number (two or three) of polarizing F substituents on the M centers compared to atoms elsewhere in the main group. Overall, therefore, the escalation in *V*_s,max_(I) for groups 13 and 14 arises from a convergence of favorable factors that may be summed up as a growing intensification of the effective electronegativity of the M centers in the fluorinated molecules, due to the substantial and unchallenged polarization of M by F substituents as M gets larger after period 3. As one reviewer points out, the polarizability of atoms below period 2 can have a big influence on other weak interactions as well, appearing, for example, to strengthen R–H···S and R–H···Se hydrogen bonds compared to analogous R–H···O bonds in certain cases.^73,74^

The sigma hole on I is a local feature of the ESP, while any computed point charge is a global value for the charge associated with the whole basin for the atom in the molecule. Yet, the natural bond orbital point charges for iodine (*q*_I(R_*n*_M–I)_) for R = H and F for periods 2 and 6 (Figure S5) prove instructive in line with the model presented in Figure 5. Going from M = Li to F in period 2, *q*_I(R_*n*_M–I)_ increases (becomes more positive) continuously, as might be expected, for both the hydrides and the fluorides. For the period 6 fluorides, however, an anomalous jump—a substantial shift to less negative point charges—is observed (going from M = Cs to At) precisely at groups 13 and 14 (F_2_Tl–I and F_3_Pb–I) consistent with a substantial F_*n*_M ← I polarization and the exceptionally positive sigma holes at I in those compounds (Figure S5). Indeed, *q*_I(F_*n*_Pb–I)_ is more positive than both *q*_I(F_*n*_Bi–I)_ and *q*_I(F_*n*_Po–I)_ where lone pair localization and charge redistribution can offset the inductive influence of the F substituents. A subtler hop is actually observed at H_2_Tl–I and H_3_Pb–I for the period 6 hydrides as well relative to the trend of the other period 6 H_*n*_M–I data points (see Figure S5), but that shift is too weak to disrupt the monotonous increase in *q*_I(H_*n*_M–I)_ from left (M = Cs) to right (M = At) in Figure S5.

### Induced Escalation: A Closer Look at Groups 13 and 14

Although electronegativities often decrease going down groups in the periodic table, that is not always the case. The Pauling electronegativities (χ_P_) for the group 14 elements vary erratically,^75,76^ and so too do their Mulliken valence state (*sp*^3^) electronegativities (χ_M_^vs^) (see values in Table S5). Indeed, Si and Sn have the lowest electronegativities in both cases, with C having the highest electronegativities, Pb second, and Ge third. As you will also see in Table S5, that kind of dramatic vacillation in electronegativities is found nowhere else in the main group—except in group 13! On both scales, Al and In are the least electronegative elements in group 13, B is the most electronegative, and Ga and Tl follow, although the ordering of the latter two differs between χ_P_ and χ_M_^vs^. And that strong showing from Ga and Tl in group 13 and Ge and Pb in group 14 (see Table S5) is thanks, in part, to the arrival of *d*-block and lanthanide contractions (between groups 2 and 13) in periods 4 and 6, respectively. Ultimately, those effects amplify nuclear charge without corresponding increases in atomic radii (enhancing, thus, the electronegativity of elements in periods 4 and 6) relative to their group members in periods 3 and 5 (Table S5).

As is clear from Figure 2a, the erratic variations in χ(M) (Table S5) are not reflected in the trends in *V*_s,max_(I) for the group 13 and 14 hydrides; *V*_s,max_(I)—as we noted before—decreases monotonously for all of the hydrides–those two groups included. But substituting for F in R_*n*_M–I polarizes the M center so disproportionately as we go down groups 13 and 14—after period 3 and the insertion of the d-block—that the effective electronegativity, χ_eff_(M), for the hybrid orbital on M to which the I atom is bonded in the M–I bond becomes apparently quite high. That climb in *χ*_eff_(M) in the M–I bond registers at I in the group 13 and 14 F_*n*_M–I molecules as jumps in *V*_s,max_(I) after period 3, especially as we go from period 3 to 4 and from period 5 to 6 (see Figure 2b). The electronegativities in Table S5, which vacillate a bit, do not actually portend a monotonous increase in *V*_s,max_(I) from periods 3 to 6, but *χ*_eff_(M) (for the M orbital in the M–I bond) in the very polarized F_*n*_M–I molecules are apparently greater and increase even more uniformly going from period 3 to period 6 (for groups 13 and 14) than even the canonical χ_P_(M) and χ_M_^vs^(M) values (Table S5) would predict.

And Bent’s rule^77^ offers some useful insights here: The pure n*s* electronegativities are necessarily larger than the n*p* electronegativities since the *s* orbitals are lower in energy, and the contribution of the n*p* orbitals to the M hybrid orbitals involved in the M–F bonds will be much richer in *p*-composition (Bent’s rule) since F is much more electronegative than I, so the hybrid orbital in the M–I bond is necessarily richer in *s*-composition and is (locally, within the bond), therefore, more electronegative than even the nominal *sp*^3^ χ_M_^vs^ values would suggest.

Why do these anomalies not appear in the hydrides? Iodine is more electronegative than the H substituent and most of the M atoms themselves, so no substantial inductive polarization of I (by the H_*n*_M– fragment) is expected. Additionally, for the reason just mentioned, and by Bent’s rule, it is the hybrid orbital to which the iodine is bonded in this case that is expected to have the greater n*p* contribution and thus the lower orbital electronegativity from M. A stepwise amplification of the sigma hole on I in H_(3–*m*)_F_*m*_M–I going from *m* = 0 to 3 has been mapped in detail for the group 14 systems^40^ and a similar progression is expected for group 13.

**Figure 6:**
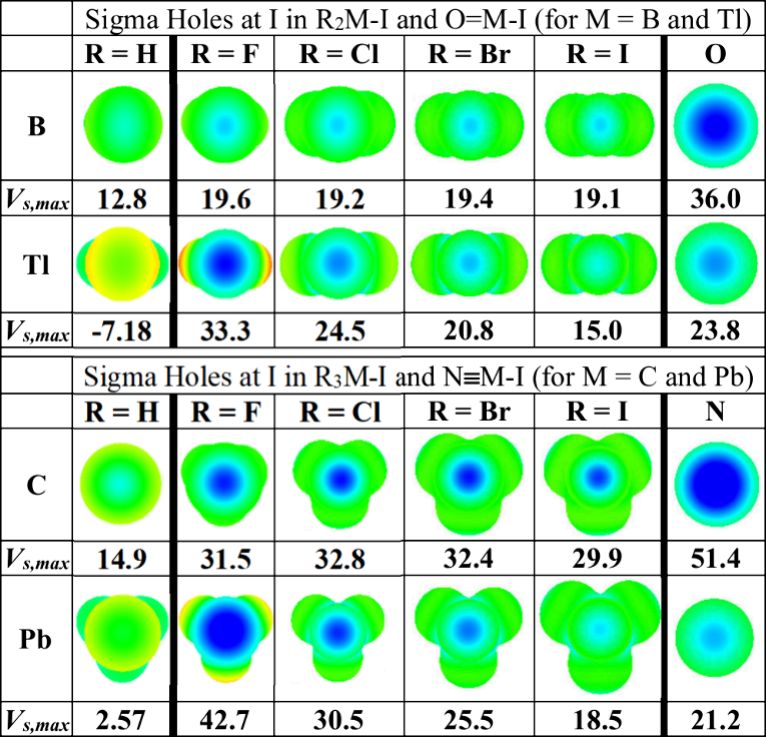
Electrostatic potentials (ESPs) mapped (in the range ±5.317 × 10^–2^ au) onto the 0.001 au isodensity surface for R_*n*_M—I monoiodides for M = B and Tl and M = C and Pb and R = H, F, Cl, Br, and I. The group 13 O=M—I and group 14 N≡M—I cases are included as well.

The presence of any drastic electronegativity (hence, *V*_s,max_) escalation in the related group 13 and 14 R_*n*_M—I compounds for R = Cl, Br, and I has not been assessed before, so we have summarized in Figure 6 our computed ESP maps and *V*_s,max_ data for the period 2 and 6 elements in those groups—B and Tl and C and Pb—for their neutral saturated H_*n*_M—I and R_*n*_M—I compounds. We considered, as well, the linear O=M—I and N≡M—I compounds (for groups 13 and 14, respectively). The latter triatomic systems offer some insight into the impact on *V*_s,max_(I) of a single strong electron withdrawing group on M but without a lone pair on that M center.

Unsurprisingly, the computed *V*_s,max_(I) values for the hydrides are relatively low (Figure 6). For the period 2 (boron and carbon) haloiodides, the *V*_s,max_(I) values change surprisingly slowly and randomly going from R = F to R = I (Figure 6), all in the range 19.3 ± 3.0 kcal·mol^–1^ for R_2_B–I and 31.5 ± 1.5 kcal·mol^–1^ for R_3_C–I, respectively. The ranges are wider for the heavier (Tl and Pb) analogues, however, and in those cases, *V*_s,max_(I) decreases briskly and monotonously in Figure 6 (going from R = F to R = I) as the R substituents become less electronegative.

Comparing the period 2 and 6 halides (Figure 6), we find that the *V*_s,max_(I) escalation observed for the fluorides—with *V*_s,max_(I) for F_2_Tl–I ≫ *V*_s,max_(I) for F_2_B–I and *V*_s,max_(I) for F_3_Pb–I ≫ *V*_s,max_(I) for F_3_C–I—persists in a weaker form for R = Cl and Br for group 13, but it disappears after the fluorides for group 14. In the latter case, the exceptionally large *V*_s,max_(I) value at F_3_PbI falls by ∼25% on going from R = F to R = Cl (see Figure 6) such that the *V*_s,max_(I) values for Cl_3_C–I and Cl_3_Pb–I are comparable. And the decline for M = Pb continues thereafter, such that the carbon *V*_s,max_(I) values are larger than the corresponding Pb values for R = Br and R = I.

For all four central M atoms considered in Figure 6, the structurally simplest and arguably most complicated electronically—namely, the group 13 oxides and group 14 nitrides—return exceptionally positive sigma holes at I for the period 2 elements (with *V*_s,max_(I) = 36.0 kcal·mol^–1^ for O=B—I and 51.4 kcal·mol^–1^ for N≡C—I) but much less impressive potentials for the heavier analogues (Figure 6). So, despite the relatively strong electronegativity of O and N as elements in the periodic table, the single polar σ bond (combined with one or two π bond(s)) does not lead to the superpolarization of the heavy group 13 and 14 M atoms in the O=M—I or N≡M—I system that is observed in some of the saturated (tri- and tetra-valent) R_*n*_M—I systems in Figure 6.

### Competing Options: A Plurality of Sigma Holes

**Figure 7:**
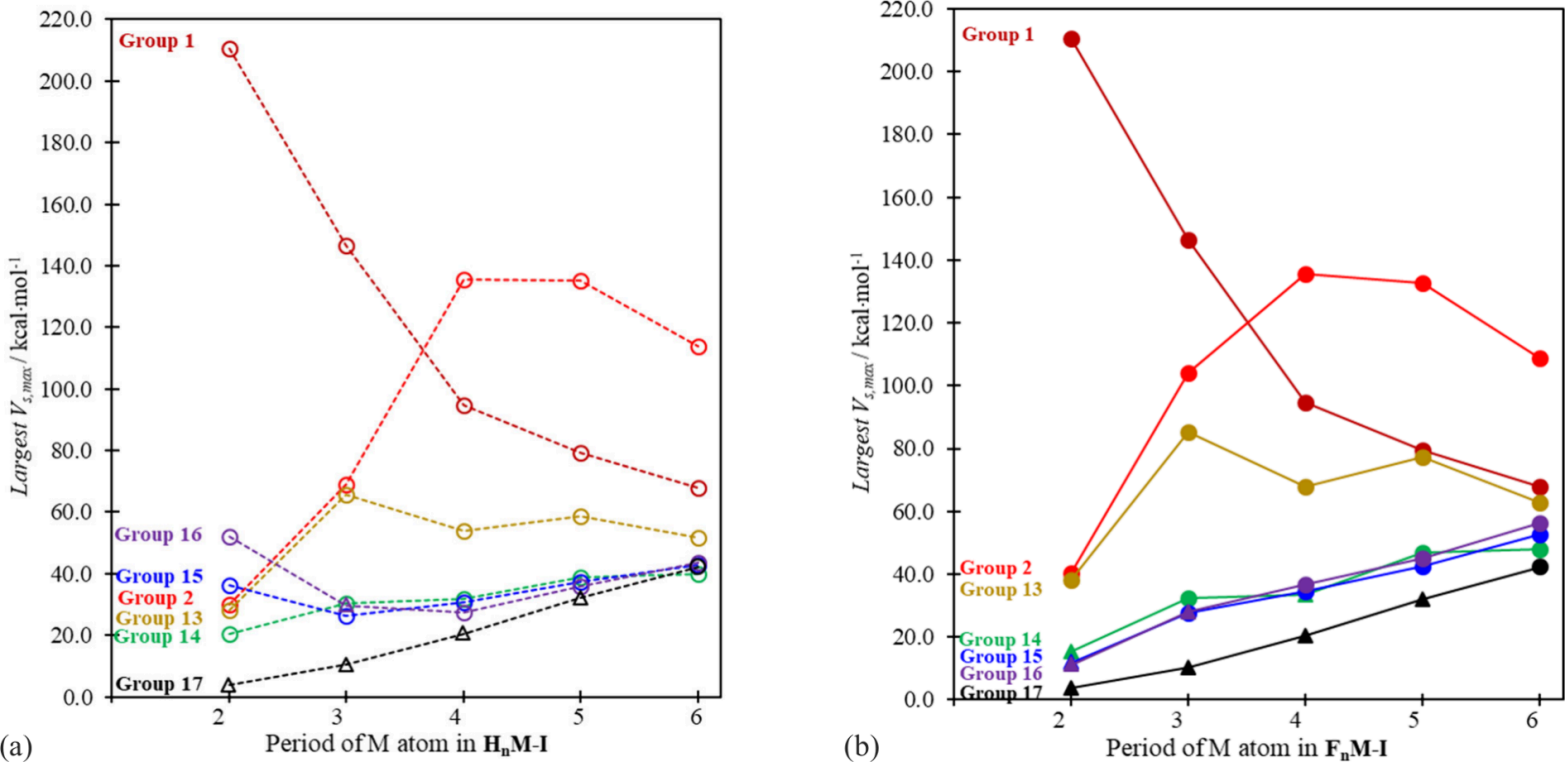
Plots of the most positive *V*_s__,__m__a__x_ values other than *V*_s,max_(I) on the 0.001 au surface of R_*n*_MI molecules (Table S14). Triangle markers identify cases where the non-*V*_s,max_(I) extremum is weaker than that of *V*_s,max_(I). The non-*V*_s,max_(I) extrema are not all of the same type. The location and nature of each is as follows (see Table S6): **Group 1**: “•M–R” type extrema for M = Li and Na and “◦M” for M = K, Rb, and Cs. **Group 2**: “Ⓜ” type extremum for M = Be, Mg, and Ca and “•M (in plane)” for M = Sr and Ba. **Group 13**: “•M⊥” in each case. **Group 14**: C–H•, “•M–I” for all other cases, except the three softest MF_3_I species (M = Ge, Sn, and Pb) where “•M–F” dominates. **Group 15**: N–H• for NH_2_I; •N–F for NF_2_I; “•M–I” for all other hydrides and PF_2_I, and “•M–F” for the three softest group 15 fluorides. Only for NF_2_I is *V*_s,max_(I) the most positive extremum. Note: We also find very weak positive regions with low *V*_s,max_ values opposite lone pair regions in group 15 and 16 fluorides and BiH_2_I. **Group 16**: “M–H•” for M = O and S (although *V*_s,max_(I) is larger for M = S), “•M–I” for the three heaviest hydrides, and “•M–F” for all fluorides. **Group 17**: “M⊙I” for M = F, Cl, Br, and I (all lower than *V*_s,max_(I)) and I–At• (which is larger than *V*_s,max_(I)).

In many cases, sigma holes are induced elsewhere (other than on the pole of the iodide atom) in the molecules considered in this work. Indeed, the maximum potentials in one or more of those regions are sometimes comparable to or larger than *V*_s,max_(I). The sigma hole on the I center is decisive for halogen bonding interactions—even if stronger sigma holes are present elsewhere in more sterically hindered parts of the molecule (such as on the central atom). Nonetheless, we wanted to achieve here a more comprehensive survey of the presence and nature of sigma holes (and ESP maxima in general) on the molecules in question, and our results showing the range of sigma holes on the isosurfaces of the R_*n*_M–I hydrides and fluorides are summarized in the Supporting Information (Tables S6–S13). For each of the main group hydride and fluoride systems under consideration, we plotted in Figure 7 the value of the largest non-*V*_s,max_(I) ESP maximum anywhere on the isodensity surface. For cases where that *V*_s,max_ value is larger than *V*_s,max_(I), a circle marker—unshaded for the hydrides and shaded for the halides—is used in Figure 7. Otherwise, *V*_s,max_(I) is the largest ESP maximum on the surface and the *V*_s,max_ value (triangle marker) included in Figure 7 is second in magnitude to *V*_s,max_(I). The actual values of the non-*V*_s,max_(I) potentials plotted in Figure 7 are listed in Table S14.

The locations of those other (non-iodine-based) positive extrema on the molecular surface vary widely even though all of the molecules share the same (R_*n*_M–I) general formula. We will employ here, therefore, a set of symbolic representations to describe the position and nature of the various sigma hole type regions in all of the R_*n*_M–I molecules under discussion (see Table S6).

For a given M–R bond, *V*_s,max_ is usually associated with one of the atoms (M or R) involved, and the extremum (*V*_s,max_) is often at the center of the sigma hole (on M or R) along the extension of the M–R bond, and we use the symbol “•M–R” or “M–R•” here to represent that familiar scenario: *V*_s,max_(I) in F–I, for instance, would be “F–I•”. Positive extrema of other sorts arise, however, on the surfaces of some of the R_*n*_M–I molecules (in groups 1, 2, 13, and 17, in particular; see Tables S6–S13 and Figures S6–S10). A ring extremum (a *V*_s,max_ ring) may arise on the outer hemisphere of a terminal atom around the pole opposite the bonding region, like a latitude line around the atom, rather than at the pole (see Figure S6). That type of ring extremum is present, for example, on the K, Rb, and Cs atoms in the group 1 M–I molecules, and such circular ESP maxima on atoms in molecules are represented here as “**◦**R”. A different form of *V*_s,max_ ring or belt extremum (abbreviated here as Ⓜ) arises around M in linear group 2, R–M–I, molecules (Figure S7). And that symmetric Ⓜ belt is distorted in such a way in the bent group 2 molecules that, instead of a ring maximum in the surface ESP around M, *V*_s,max_ is localized at a spot on M (denoted “•M (in plane)”) that is in the plan of the bent R–M–I molecule, opposite the direction of the bending (Figure S8).

In the trigonal planar group 13 dihalides, a positive extremum appears on the central M atom at the only points where the central atom is substantially exposed—i.e., perpendicular to the molecular plane—and, for that case, *V*_s,max_ is denoted “**•M⊥**” (see Figure S9). The (group 17) haloiodides have a belt of positive potentials around the M–I bond (not on either of the two atoms), and that feature is represented here as “**M⊙R**” (see Figure S10). These symbols (which are described, with illustrations as well, in the Supporting Information) are used in the caption to Figure 7 to describe the nature of the largest *V*_s,max_ values (other than *V*_s,max_(I)) on the isosurfaces of each of the molecules (see also Table S14).

The various bond- or atom-centered positive extrema that compete with *V*_s,max_(I) in the R_*n*_M–I molecules are intriguing. The largest *V*_s,max_ value identified in any of the structures in Figure 7 is that on Li in Li–I (210.6 kcal·mol^–1^). And those induced on M in the groups 1 and 2 compounds are consistently stronger than the corresponding *V*_s,max_(I). The iodine-centered sigma hole extremum (*V*_s,max_(I)) becomes more competitive relative to all of those other sigma hole sites and starts to dominate as we move from group 1 to group 17 in Figure 7 (see Table S14). But even in the cases of the group 13 and 14 heavy fluoroiodides, the sigma holes induced on M (the lures for triel and tetrel bonding) are somewhat stronger than *V*_s,max_(I) in most cases, except for CF_3_I and GeF_3_I.

Yet, although the sigma hole at M, for instance, can be quite strong, terminal atom (especially H and halogen) sigma holes are crucial molecular features to assess and understand. Terminal atoms usually suffer much less from steric hindrance than central atoms, so even in cases where they are somewhat weaker than other positive extrema on a molecular surface, their relative accessibility can allow them to dominate intermolecular interactions. Central atom sigma hole strengths are controlled by the identity of substituents, and thanks to inductive effects, halogen atom sigma hole strengths (hence halogen bonding) can be tuned over a very broad range as well by the strategic selection of both the central atom involved and other (geminal) substituents.

## Conclusions

The possibilities for viable halogen bonding interactions by halogenated heavy atoms have been largely ignored on both the computational and experimental fronts. Yet we know that in some cases, depending on the identity of R, the I atom in the R_3_Ge–I, R_3_Sn–I, and R_3_Pb–I type molecules with heavier group 14 central atoms can exhibit sigma holes and form halogen bonds that are larger and stronger than those of their carbon analogue.^40,42^

As part of a comprehensive assessment of halogen bonding by main group R_*n*_M–I systems (for M = H to At, excluding group 18), we have reconfirmed in this work what may be described as the superpolarization of the heavy group 14 M atoms, in R_3_M–I compounds when R = F and an associated escalation in the magnitude of the positive potential at the sigma hole generated on I. More significantly, we find that this sigma hole escalation phenomenon is not isolated to group 14 compounds or to fluorides. The same anomalous behavior is observed in certain saturated group 13 R_2_M–I type molecules, but it disappears as we move beyond group 14 to group 15, 16, and 17 main group M atoms. And a model is provided here to explain this phenomenon.

A detailed investigation of the distribution of positive (sigma hole type) extrema on the surfaces of these model R_*n*_M–I systems has been carried out as well, and the strengths of those various types of “holes” on the molecular isosurface relative to the maximum electrostatic potential at the iodine sigma hole have been quantified for the whole series of main group compounds.

Other cases in which the sigma hole escalation phenomenon might be observed in R_*n*_M–I molecules (where R ≠ H or F) are examined and discussed. The potentially significant utility of halogen and other sigma hole bonds by halides of heavy main group atoms has been demonstrated. Several compounds of those elements appear to be able to pull their weight, as it were, as bonding partners in forming strong non-covalently bonded complexes.

## Supporting Information Available

Interaction energy data and graphs, descriptions and examples of surface electrostatic potential maps, tables summarizing electronegativities and different types of extrema on the R_*n*_M–I potential surfaces, complete Gaussian 16 reference, and sources for the several core potentials and basis sets used in this work (PDF)
A set of viewable Cartesian coordinates for systems studied (a folder of “.xyz” files) (ZIP)
