# Supplementary material for: Polarization Upends Convention: Halogen Bonding Propensities of Main Group Halides
Source: J Phys Chem A. 2025 Jan 15;129(4):955–66. doi: 10.1021/acs.jpca.4c06456 (PMC11789142; doi:10.1021/acs.jpca.4c06456)
Supplement: Supplementary file 1 — jp4c06456_si_001.pdf [file jp4c06456_si_001.pdf]

# Polarization Upends Convention: Halogen Bonding Propensities of Main Group Halides

Noah Robinson, Nam Pham, and Kelling J. Donald,*

Department of Chemistry, Gottwald Center for the Sciences, University of Richmond, Richmond, Virginia 23173, United States

## Supporting Information

* Corresponding author. K. J. Donald, Tel.: 1-804-484-1628. E-mail: kdonald@richmond.edu ORCID: 0000-0001-9032-4225

**Table of Contents** (Tables and figures are arranged in the order mentioned in main text.)


| Abbreviated Captions[1] | Page |
|---|---|
| Guide to *.xyz* Files | S3 |
| **Table S1**: $V_{s,max}$(I), in kcal·mol$^{-1}$ units, for hydrides and fluorides considered. | S3 |
| **Table S2**: BSSE corrected halogen bonding interaction energies, ΔE, in kcal·mol$^{-1}$ units. | S3 |
| **Table S3**: BSSE corrected ΔH(298.15K), in kcal·mol$^{-1}$ units, for halogen bonds. | S4 |
| **Table S4**: BSSE corrected ΔG(298.15K), in kcal·mol$^{-1}$ units, for halogen bonds. | S4 |
| **Table S5:** Pauling and Mulliken Valence State Electronegativities | S4 |
| **Table S6**: Definitions and symbolic representations of other positive extrema on the surface of the $MH_nI$ and $MF_nI$ molecules. | S5 |
| **Table S7**: Group 1: $V_{s,max}$ values in kcal·mol$^{-1}$ units (other than $V_{s,max}$(I)) for MI. | S5 |
| **Table S8**: Group 2: $V_{s,max}$ values in kcal·mol$^{-1}$ units (other than $V_{s,max}$(I)) for MRI. | S6 |
| **Table S9**: Group 13: $V_{s,max}$ values in kcal·mol$^{-1}$ units (other than $V_{s,max}$(I)) for $MR_2I$. | S6 |
| **Table S10**: Group 14: $V_{s,max}$ values in kcal·mol$^{-1}$ units (other than $V_{s,max}$(I)) for $MR_3I$. | S7 |
| **Table S11**: Group 15: $V_{s,max}$ values in kcal·mol$^{-1}$ units (other than $V_{s,max}$(I)) for $MR_2I$. | S7 |
| **Table S12**: Group 16: $V_{s,max}$ values in kcal·mol$^{-1}$ units (other than $V_{s,max}$(I)) for MRI. | S8 |
| **Table S13**: Group 17: $V_{s,max}$ values in kcal·mol$^{-1}$ units (other than $V_{s,max}$(I)) for MI. | S8 |
| **Table S14**: Most positive $V_{s,max}$ values apart from the $V_{s,max}$(I) value. | S9 |
| **Figure S1**: The sigma hole on I in the H-I molecule presented in contrast with the sigma hole on I in the group 17 M-I molecules. | S10 |
| **Figure S2**: Plot of BSSE corrected computed interaction energies, ΔE, in kcal·mol$^{-1}$ units, for halogen bonding interactions. | S10 |
| **Figure S3**: Plot of BSSE corrected computed enthalpy changes, ΔH(298.15K), in kcal·mol$^{-1}$ units, for halogen bonding interactions. | S10 |
| **Figure S4**: Plot of BSSE corrected computed free energy changes, ΔG(298.15K), in kcal·mol$^{-1}$ units, for halogen bonding interactions. | S11 |
| **Figure S5**: Computed Natural Bond Orbital (NBO) point charges for I in selected $R_nM$-I molecules | S11 |
| **Figure S6**: ESP iso-surface showing positive $V_{s,max} \neq V_{s,max}$(I) [Case: ○M; terminal atom ring $V_{s,max}$] | S12 |
| **Figure S7**: ESP iso-surface showing positive $V_{s,max} \neq V_{s,max}$(I) [Case: Ⓜ; central atom ring $V_{s,max}$] | S12 |
| **Figure S8**: ESP iso-surface showing positive $V_{s,max} \neq V_{s,max}$(I) [Case: •M (in plane); central atom point $V_{s,max}$] | S12 |
| **Figure S9**: ESP iso-surface showing positive $V_{s,max} \neq V_{s,max}$(I) [Case: •M ⊥; central atom perpendicular $V_{s,max}$] | S13 |
| **Figure S10**: ESP iso-surface showing positive $V_{s,max} \neq V_{s,max}$(I) [Case: M⊙R; bond ring $V_{s,max}$] | S13 |
| Specific references (sources) for Stuttgart-Cologne ECPs and basis sets used in this work. | S14 |
| Complete Gaussian 16 Reference | S14 |


[1] More detailed captions are provided with the individuals figures and tables.

## Guide to .xyz Files:

The linked folder contains .xyz files with optimized coordinates for the isolated groups 13 and 14 iodohydrides and iodohalides, group 17 iodides, the two bases ($NH_3$, and $N(CH_3)_3$), and pairwise complexes formed by the iodides and each of the bases.

## TABLES

**Table S1**: Maximum potential in the sigma hole on the I atom, $V_{s,max}$(I), in kcal·mol$^{-1}$ units, for $MH_nI$ and $MF_nI$. These data are plotted in graphs in the main text.

| | **M Group (for Hydrides - $MH_nI$)** | | | | | | |
|---|---|---|---|---|---|---|---|
| **Period** | **1[(a)]** | **2** | **13** | **14** | **15** | **16** | **17[(a)]** |
| **2** | -28.0 | 7.8 | 12.8 | 14.9 | 26.6 | 40.4 | 57.7 |
| **3** | -35.6 | -4.4 | 4.8 | 10.7 | 16.0 | 30.1 | 46.6 |
| **4** | -42.6 | -16.1 | 3.8 | 8.8 | 12.4 | 25.5 | 40.9 |
| **5** | -44.3 | (b) | 0.0 | 6.0 | 7.6 | 18.7 | 32.1 |
| **6** | (b) | (b) | -7.2 | 2.6 | 3.4 | 14.2 | 27.0 |
| | **M Group (for Fluorides - $MF_nI$)** | | | | | | |
| **Period** | **1[(a)]** | **2** | **13** | **14** | **15** | **16** | **17[(a)]** |
| **2** | | 10.6 | 19.6 | 31.5 | 39.4 | 51.8 | |
| **3** | | 0.61 | 17.1 | 24.9 | 16.5 | 32.9 | |
| **4** | | -17.5 | 24.6 | 33.5 | 16.6 | 28.9 | |
| **5** | | (b) | 25.2 | 35.3 | 13.1 | 22.3 | |
| **6** | | (b) | 33.3 | 42.7 | 10.1 | 18.3 | |

(a) Same values for both sections of the table since $n = 0$ for groups 1 and 17. For the H-I molecule, $V_{s,max}$(I) = 23.6 kcal·mol$^{-1}$.
(b) No positive maximum was identified on the I center in any of these compounds. In CsI, SrHI, SrFI, BaHI, and BaFI there is in fact a negative extremum, $V_{s,min}$, on I instead.

**Table S2**: BSSE corrected computed interaction energies, ΔE, in kcal·mol$^{-1}$ units, for the $H_nM$-I---Base, and $F_nM$-I---Base complexes, where M is a main group atom and Base = $NH_3$, or $N(CH_3)_3$.

| | **Hydrides** | | | | | | **Fluorides** | | | | | |
|---|---|---|---|---|---|---|---|---|---|---|---|---|
| | **Base = $NH_3$** | | | **Base = $N(CH_3)_3$** | | | **Base = $NH_3$** | | | **Base = $N(CH_3)_3$** | | |
| **Period** | **Gp. 13** | **Gp. 14** | **Gp. 17** | **Gp. 13** | **Gp. 14** | **Gp. 17** | **Gp. 13** | **Gp. 14** | **Gp. 17[(b)]** | **Gp. 13** | **Gp. 14** | **Gp. 17[(b)]** |
| **2** | -2.02 | -3.10 | -17.86 | -3.18 | -4.75 | -22.41 | -3.04 | -6.24 | -17.86 | -4.21 | -8.04 | -22.41 |
| **3** | -1.09 | -2.06 | -13.73 | -2.25 | -3.37 | -18.13 | -2.53 | -3.93 | -13.73 | -3.58 | -5.06 | -18.13 |
| **4** | -1.12 | -1.98 | -11.83 | -2.38 | -3.41 | -15.96 | -3.94 | -5.83 | -11.83 | -5.12 | -7.27 | -15.96 |
| **5** | -0.75 | -1.66 | -8.88 | -2.10 | -3.09 | -12.36 | -4.22 | -6.46 | -8.88 | -5.57 | -8.28 | -12.36 |
| **6** | (a) | -1.47 | -7.54 | -1.67 | -3.05 | -10.75 | -6.65 | -10.44 | -7.54 | -8.93 | -15.00 | -10.75 |

(a) No $H_2Tl$-I---$NH_3$ complex was obtained. A weak hydride complex was formed with the stronger ($N(CH_3)_3$) base.
(b) The *hydride* and *fluoride* values for group 17 are identical since group 17 M-I molecules have no M-H or M-F bond.

**Table S3**: BSSE corrected computed enthalpy changes, ΔH(298.15K), in kcal·mol$^{-1}$ units, for the $H_nM$-I---Base and $F_nM$-I---Base complexes, where M is a main group atom and Base = $NH_3$, or $N(CH_3)_3$.

| | Hydrides | | | | | | Fluorides | | | | | |
|---|---|---|---|---|---|---|---|---|---|---|---|---|
| | Base = $NH_3$ | | | Base = $N(CH_3)_3$ | | | Base = $NH_3$ | | | Base = $N(CH_3)_3$ | | |
| **Period** | **Gp. 13** | **Gp. 14** | **Gp. 17** | **Gp. 13** | **Gp. 14** | **Gp. 17** | **Gp. 13** | **Gp. 14** | **Gp. 17[b]** | **Gp. 13** | **Gp. 14** | **Gp. 17[b]** |
| **2** | -0.78 | -1.80 | -15.56 | -1.88 | -3.41 | -19.21 | -1.72 | -4.68 | -15.56 | -2.86 | -6.29 | -19.21 |
| **3** | 0.13 | -0.78 | -11.51 | -1.02 | -2.06 | -14.87 | -1.24 | -2.54 | -11.51 | -2.26 | -3.63 | -14.87 |
| **4** | 0.10 | -0.72 | -9.86 | -1.16 | -2.12 | -13.05 | -2.56 | -4.29 | -9.86 | -3.68 | -5.56 | -13.05 |
| **5** | 0.57 | -0.43 | -7.29 | -0.78 | -1.82 | -10.06 | -2.80 | -4.85 | -7.29 | -4.07 | -6.36 | -10.06 |
| **6** | [a] | 0.11 | -6.09 | -0.50 | -1.50 | -8.75 | -5.06 | -8.30 | -6.09 | -6.95 | -11.53 | -8.75 |

[a] No $H_2Tl$-I---$NH_3$ complex was obtained. A weak hydride complex was formed with the stronger ($N(CH_3)_3$) base.
[b] The *hydride* and *fluoride* values for group 17 are identical since group 17 M-I molecules have no M-H or M-F bond.

**Table S4**: BSSE corrected computed free energy changes, ΔG(298.15K), in kcal·mol$^{-1}$ units, for the $H_nM$-I---Base and $F_nM$-I---Base complexes, where M is a main group atom and Base = $NH_3$, or $N(CH_3)_3$.

| | Hydrides | | | | | | Fluorides | | | | | |
|---|---|---|---|---|---|---|---|---|---|---|---|---|
| | Base = $NH_3$ | | | Base = $N(CH_3)_3$ | | | Base = $NH_3$ | | | Base = $N(CH_3)_3$ | | |
| **Period** | **Gp. 13** | **Gp. 14** | **Gp. 17** | **Gp. 13** | **Gp. 14** | **Gp. 17** | **Gp. 13** | **Gp. 14** | **Gp. 17[b]** | **Gp. 13** | **Gp. 14** | **Gp. 17[b]** |
| **2** | 5.18 | 5.17 | -6.28 | 5.36 | 4.52 | -8.74 | 4.66 | 3.02 | -6.28 | 3.72 | 2.22 | -8.74 |
| **3** | 4.31 | 4.91 | -2.60 | 4.22 | 4.89 | -4.78 | 4.70 | 3.61 | -2.60 | 4.44 | 3.56 | -4.78 |
| **4** | 4.70 | 4.56 | -1.18 | 4.21 | 4.49 | -3.14 | 3.92 | 2.11 | -1.18 | 3.96 | 2.45 | -3.14 |
| **5** | 5.09 | 4.67 | 0.59 | 5.61 | 5.19 | -0.97 | 3.78 | 2.52 | 0.59 | 3.87 | 2.14 | -0.97 |
| **6** | [a] | 6.13 | 2.10 | 6.30 | 6.46 | 0.58 | 1.90 | -0.11 | 2.10 | 2.21 | -1.93 | 0.58 |

[a] No $H_2Tl$-I---$NH_3$ complex was obtained. A weak hydride complex was formed with the stronger ($N(CH_3)_3$) base.
[b] The *hydride* and *fluoride* values for group 17 are identical since group 17 M-I molecules have no M-H or M-F bond.

**Table S5**: Pauling ($\chi_P$) and Mulliken valence state ($\chi_M^{vs}/eV$) electronegativities.

| [a,b] **Pauling Electronegativities** ($\chi_P$) | | | | | | | [a,c] **Mulliken (valence state) Electronegativities** ($\chi_M^{vs}$) | | | | | | |
|---|---|---|---|---|---|---|---|---|---|---|---|---|---|
| | | | | | | | ***s*** | ***sp*** | ***sp*$^2$** | ***sp*$^3$** | [c]***sp*$^4$** | [c]***sp*$^5$** | [c]***sp*$^6$** |
| Li | Be | B | C | N | O | F | Li | Be | B | C | N | O | F |
| 0.98 | 1.57 | 2.04 | 2.55 | 3.04 | 3.44 | 3.98 | 3.005 | 4.65 | 6.37 | 8.15 | 10.00 | 12.55 | 15.30 |
| Na | Mg | Al | Si | P | S | Cl | Na | Mg | Al | Si | P | S | Cl |
| 0.93 | 1.31 | 1.61 | 1.90 | 2.19 | 2.58 | 3.16 | 2.844 | 4.11 | 5.61 | 7.30 | 7.41 | 9.04 | 10.95 |
| K | Ca | Ga | Ge | As | Se | Br | K | Ca | Ga | Ge | As | Se | Br |
| 0.82 | 1.00 | 1.81 | 2.01 | 2.18 | 2.55 | 2.96 | 2.421 | 3.29 | 6.28 | 7.53 | 7.25 | 8.65 | 10.25 |
| Rb | Sr | In | Sn | Sb | Te | I | Rb | Sr | In | Sn | Sb | Te | I |
| 0.82 | 0.95 | 1.78 | 1.96 | 2.05 | (2.1)[d] | 2.66 | 2.332 | 3.07 | 5.39 | 7.05 | 6.68 | 7.83 | 9.29 |
| Cs | Ba | Tl | Pb | Bi | Po | At | Cs | Ba | Tl | Pb | Bi | Po | At |
| 0.79 | 0.89 | 2.04 | 2.33 | 2.02 | (2.0)[d] | (2.2)[d] | 2.183 | 2.79 | 6.08 | 7.82 | 6.81 | 8.14 | 9.76 |

[a]For hydrogen, the relevant values are: $\chi_P$(H) = 2.20 and $\chi_M^{vs\,(s)}$(H) = 7.176 eV. [b]Refs. 1, and 2. [c]Ref. 1; *ad hoc* estimates of *s-p* hybridization – $sp^4$ ≡ 20%, $sp^5$ ≡ 16.7%, $sp^6$ ≡ 14.3% – derived using a simple formula (%*s* = 100/n, where n = 1,2,…7, going from group 1 to 17 in the main group) which allows for a systematic increase in the *p* contribution in the bond (and a corresponding increase in the *s* contribution to the lone pair(s)) without resorting to the extremes ($sp^3$ hybridization for bonds and lone pairs or 100% *p* in the bonds) starting at group 15. [d]These values were not included in Refs. 1 or 2; they are from Ref. 3.


**References (for Table S5 above)**

(1) Bratsch, S. G. Revised Mulliken Electronegativities: I. Calculation and Conversion to Pauling Units. *J. Chem. Educ.* **1988**, *65*, 34-41.
(2) Allred, A. L. Electronegativity Values from Thermochemical Data *J. Inorg. Nucl. Chem.* **1961**, *17*, 215-221.
(3) Huheey, J. E. Inorganic Chemistry: Principles of Structure and Reactivity 3rd ed.; Harper & Row: New York, 1983.

**Table S6**: Definitions and symbols for various types of positive extrema, $V_{s,max}$, on the surface of $MH_nI$ and $MF_nI$ molecules. The definitions apply for any molecule with an 'M-R' bond. M and R may be any element, but we assume that R is a terminal atom and M can be a terminal or central atom.

| Symbol | Definition |
|---|---|
| •M-R or M-R• | [Point extremum (on M or R) opposite the M-R bond] '•M-R' denotes the maximum of the sigma hole on M located, as is typical, *at the center of the sigma hole* (at the pole of M, i.e. opposite the M-R bond along the bond extension). For an analogous sigma hole on R in that molecule, the symbol would be M-R•. |
| ○M | [Ring extremum (on atom surface)] This refers to a less common case where a ring extremum arises as a circle in the sigma hole region opposite a bond. In this case, the extremum is not a point but a ring *around the extension of the bond axis*, e.g. on K, Rb, and Cs in K-I, Rb-I, and Cs-I, respectively. In such instances, the potential usually increases slightly as you radiate out from the pole, reaching a maximum along the way and decreasing again as you move toward the equator. See Figure S6. |
| Ⓜ | [Ring extremum (around central M atom)] This symbol refers to a ring extremum that arises *around M in the middle of the (usually linear) molecule*, e.g. on Be, Mg, and Ca in the triatomic R-Be-I, R-Mg-I, and R-Ca-I molecules, respectively. See Figure S7. |
| •M (in plane) | [Point extremum (in molecular plane but not associated with one specific bond)] This type of extremum arises when the relevant region of positive potential (hence its extremum) is not immediately opposite any one bond. It might arise as the net result of polarization by more than one bond in a low symmetry structure such as the bent group 2 RMI compounds in this case (i.e. for M = Sr, and Ba). The bending leads to a localization of the extremum instead of the ring extremum observed in the linear RMI molecules where M = Be, Mg, and Ca (See Figure S8). |
| •M ⊥ | [Point extremum (perpendicular to molecular plane)] This denotes a region of positive potential that arises (on the exposed surface of a very polarized central atom) perpendicular to the plane of the molecule. Sometimes called a π-hole, it is typical for group 13 molecules with trigonal planar structures (see Figure S9). |
| M⊙R | [Ring extremum (around a bond)] This refers to a ring or belt of positive potential *around a bond* between two atoms (e.g. around the M-I bond in group 17 monoiodides). See Figure S10. |

**Table S7**: **Group 1**: Local electrostatic potential (ESP) maxima, $V_{s,max}$, in kcal·mol$^{-1}$ units (other than $V_{s,max}$(I)) for group 1 M-I molecules.[(a)]

| **M** | $V_{s,max}$ **type** | $V_{s,max}$ |
|---|---|---|
| H | •H-I | 27.2 |
| Li | •Li-I | 211 |
| Na | •Na-I | 147 |
| K | ○K | 94.6 |
| Rb | ○Rb | 79.4 |
| Cs | ○Cs | 67.8 |

[(a)] For H-I, there is a sigma hole on H ($V_{s,max}$ = 27.2 kcal·mol$^{-1}$) that is a bit more positive than the sigma hole on the slightly more electronegative I atom (i.e. $V_{s,max}$ = 23.6 kcal·mol$^{-1}$). For H, Li, and Na, the extremum ($V_{s,max}$) is at the center of the sigma hole (at the pole of M opposite the M-I bond) as indicated by the dot here: •M-I. For K, Rb, and Cs, the extremum is a belt of maximum positive potential (a sigma belt), like a latitude line, in the middle of the sigma hole region (see Figure S6).

**Table S8**: **Group 2**: Local electrostatic potential (ESP) maxima, $V_{s,\max}$, in kcal·mol$^{-1}$ units (other than $V_{s,max}$(I)) for MHI and MFI.[a]

| | Hydrides | | Fluorides | |
|---|---|---|---|---|
| **M** | $V_{s,max}$ **type** | $V_{s,max}$ | $V_{s,max}$ **type** | $V_{s,max}$ |
| Be | (Be) | 30.0 | (Be) | 40.5 |
| | Be-H• | -7.6 | | |
| Mg | (Mg) | 68.9 | (Mg) | 104 |
| Ca | (Ca) | 136 | (Ca) | 136 |
| Sr | •Sr[b] | 135 | •Sr[b] | 133 |
| Ba | •Ba[b] | 114 | •Ba[b] | 109 |

[a] A positive ring or belt is observed around Be, Mg, and Ca in the linear R-M-I molecules (see Figure S7). Rather than a potential maximum, a negative extremum ($V_{s,min}$) is observed on the cap of F opposite the F-M bond in all group 2 fluorides.

**[b]** For these bent MHI and MFI molecules, part of the positive ring in the linear MRI system is lost upon bending and a localized maximum in the potential arises on M (opposite the direction of the bending) in a region roughly at the intersection of the plane of the R-M-I molecule and a perpendicular plane though M cutting through the middle of the R-M-I bond angle (see Figure S8). Of note, no sigma hole is observed on I for these bent group 2 dihalides; a negative extremum is observed on I instead.

**Table S9**: **Group 13**: Local electrostatic potential (ESP) maxima, $V_{s,max}$, in kcal·mol$^{-1}$ units (other than $V_{s,max}$(I)) for $MH_2I$ and $MF_2I$.[a]

| | Hydrides | | Fluorides | |
|---|---|---|---|---|
| **M** | $V_{s,max}$ **type** | $V_{s,max}$ | $V_{s,max}$ **type** | $V_{s,max}$ |
| B | B-H• | 6.1 | •B ⊥ | 38.3 |
| | •B ⊥ | 28.0 | | |
| Al | •Al ⊥ | 65.8 | •Al ⊥ | 85.4 |
| Ga | •Ga ⊥ | 54.0 | •Ga ⊥ | 67.9 |
| In | •In ⊥ | 58.8 | •In ⊥ | 77.2 |
| Tl | Tl-H• | 11.0 | •Tl ⊥ | 62.9 |
| | •Tl ⊥ | 51.6 | | |

[a] For the Group 13 compounds, the most positive sigma holes are a pair located on the central atom, perpendicular (above and below) the plan of the roughly trigonal planar $MR_2I$ molecule. There is a weaker sigma hole on H in $BH_2I$ and $TlH_2I$, but none for the other M atoms – Al, Ga, and In. There is no sigma hole on F in any of these compounds. See Figure S9.

**Table S10**: **Group 14**: Local electrostatic potential (ESP) maxima, $V_{s,max}$, in kcal·mol$^{-1}$ units (other than $V_{s,max}$(I)) for $MH_3I$ and $MF_3I$.[a,b]

| | Hydrides | | Fluorides | |
|---|---|---|---|---|
| **M** | $V_{s,max}$ **type** | $V_{s,max}$ | $V_{s,max}$ **type** | $V_{s,max}$ |
| C | C-H• | 20.4 | C-F• | -1.2 |
| | •C-I | 13.4 | •C-F | 14.2 |
| | | | •C-I | 15.2 |
| Si | •Si-H | 18.6 | •Si-F | 27.6 |
| | •Si-I | 30.4 | •Si-I | 32.2 |
| Ge | •Ge-H | 18.2 | •Ge-F | 33.4 |
| | •Ge-I | 31.9 | •Ge-I | 30.0 |
| Sn | •Sn-H | 23.0 | •Sn-F | 46.9 |
| | •Sn-I | 38.7 | •Sn-I | 43.2 |
| Pb | •Pb-H | 21.7 | •Pb-F | 48.1 |
| | •Pb-I | 39.9 | •Pb-I | 36.6 |

[e] The most positive extrema, or the next most, after $V_{s,max}$(I), among positive potentials on the isodensity surfaces for the group 14 molecules are those induced on H in $CH_3I$, by I on M (that is I-M•) in other hydrides, and by I or F on M for the fluorides.

[b] Note: For any central atom, M, with multiple substituents, the influence of any one substituent on M is not necessarily decoupled from the influence of others on M, since they are all withdrawing electron density from (or donating electron density to) that one center. So, the sigma hole induced on M opposite any M-R bond may be primarily (though not necessarily exclusively) due to the influence of that single R substituent; notice in the data above, for example, that $V_{s,max}$ for any •M-I in $MH_3I \neq V_{s,max}$ for the corresponding •M-I in $MF_3I$. This should be kept in mind even as we associate each R-M• sigma hole specifically with the single R-M bond (hence the R substituent) directly across from that sigma hole.

**Table S11**: **Group 15**: Local electrostatic potential (ESP) maxima, $V_{s,max}$, in kcal·mol$^{-1}$ units (other than $V_{s,max}$(I)) for $MH_2I$ and $MF_2I$.[a,b]

| | Hydrides | | Fluorides | |
|---|---|---|---|---|
| **M** | $V_{s,max}$ **type** | $V_{s,max}$ | $V_{s,max}$ **type** | $V_{s,max}$ |
| N | N-H• | 36.4 | N-F• | -1.8 |
| | | | •N-F | 11.8 |
| | | | •N-lp | 8.6 |
| | | | •N-I | 4.4 |
| P | P-H• | 15.7 | •P-F | 25.6 |
| | •P-H | 14.2 | •P-lp | 7.8 |
| | •P-I | 26.3 | •P-I | 27.6 |
| As | As-H• | (none) | •As-F | 34.5 |
| | •As-H | 16.7 | •As-lp | 3.3 |
| | •As-I | 30.7 | •As-I | 32.3 |
| Sb | Sb-H• | (none) | •Sb-F | 42.5 |
| | •Sb-H | 22.1 | •Sb-lp | 4.0 |
| | •Sb-I | 37.4 | •Sb-I | 39.5 |
| Bi | •Bi-H | 26.0 | •Bi-F | 52.7 |
| | •Bi-lp | -0.4 | •Bi-lp | 5.1 |
| | •Bi-I | 42.9 | •Bi-I | 47.9 |

[a] For the group 15 compounds, the other positive extrema (which are usually more positive than $V_{s,max}$(I)) arise typically from the polarization of M by I in the hydrides (except for M = N, where the H sites have the most positive sigma holes) or by F in the case of the fluorides (except for $PF_2I$ where the sigma hole on P opposite the P-I bond is slightly more positive. An interesting feature of these data overall is that sigma holes induced by F and by I on M in some of $MF_nI$ molecules are surprisingly close to each other in value given the significant electronegativity difference between F and I (see footnote (b) below Table S10).

[b] A Relatively weak **s**igma hole arises as well on M in the region opposite to the lone pair (near the center of the F-I-F face of the $MF_2I$ distorted trigonal pyramid) in the groups 15 fluorides and $BiH_2I$. Those sigma holes are denoted '•M-lp'.

**Table S12: Group 16**: Local electrostatic potential (ESP) maxima, $V_{s,max}$, in kcal·mol$^{-1}$ units (other than $V_{s,max}$(I)) for MHI and MFI.[a,b]

| | Hydrides | | Fluorides | |
|---|---|---|---|---|
| **M** | $V_{s,max}$ **type** | $V_{s,max}$ | $V_{s,max}$ **type** | $V_{s,max}$ |
| O | O-H• | 52.2 | O-F• | -3.7 |
| | •O-H | -4.0 | •O-F | 11.1 |
| | | | •O-I | -9.1 |
| | | | •O-lp | 4.8 |
| S | S-H• | 29.8 | S-F• | -9.8 |
| | •S-H | 9.3 | •S-F | 28.0 |
| | | | •S-I | 17.9 |
| | | | •S-lp | 2.7 |
| Se | Se-H• | 24.7 | Se-F• | -15.4 |
| | •Se-H | 14.8 | •Se-F | 36.8 |
| | •Se-I | 27.6 | •Se-I | 26.4 |
| | | | •Se-lp | 1.6 |
| Te | •Te-H | 22.2 | Te-F• | - |
| | •Te-I | 36.0 | •Te-F | 45.2 |
| | | | •Te-I | 36.5 |
| | | | •Te-lp | 2.6 |
| Po | •Po-H | 28.9 | Po-F• | - |
| | •Po-I | 43.7 | •Po-F | 56.4 |
| | | | •Po-I | 46.5 |
| | | | •Po-lp | 2.0 |

[a] In the group 16 hydrides, the competing or stronger sigma holes are the sigma holes on H (for M = O and S) and those induced by I on M, for M = Se, Te, and Po. The sigma holes induced by F on M dominate in the heavier fluorides, but they are weaker than $V_{s,max}$(I) in OFI and SFI.
[b] A Relatively weak **s**igma hole arises as well on M in the region opposite to the lone pairs (lp) in the group 16 fluorides. Those sigma holes are denoted '•M-lp'.

**Table S13**: **Group 17**: Local electrostatic potential (ESP) maxima, $V_{s,max}$, in kcal·mol$^{-1}$ units (other than $V_{s,max}$(I), except for $I_2$[a]) for group 17 M-I molecules.[b]

| **M** | $V_{s,max}$ **type** | $V_{s,max}$ |
|---|---|---|
| F | •F-I | -16.3 |
| | F⊙I | 3.9 |
| Cl | •Cl-I | 10.3 |
| | Cl⊙I | 2.0 |
| Br | •Br-I | 20.6 |
| | Br⊙I | 1.3 |
| I | •I-I | 32.1[a] |
| | I⊙I | 0.9 |
| At | •At-I | 42.2 |
| | At⊙I | 0.17 |

[a] M = I. [b] A sigma belt emerges around the M-I bond itself. That belt, M⊙I, is consistently weaker than •M-I, except when M = F. For M = At, the sigma hole induced on At by I is stronger than the sigma hole on I ($V_{s,max}$(I) = 27.0 kcal·mol$^{-1}$). The I-At molecule is expected, therefore, to form, preferentially, a I-At---Base halogen bonded complex rather than the At-I---Base system. In all other cases, $V_{s,max}$(I) is the most positive extremum on the group 17 M-I molecular surface. See Figure S10.

**Table S14**: Most positive $V_{s,max}$ values (in kcal·mol$^{-1}$), apart from $V_{s,max}$(I), on the 0.001 au surface of $R_nMI$ molecules. Values in bold are more positive than $V_{s,max}$(I) for the relevant molecule (see Table S1). The nature of each of these *non-$V_{s,max}$(I) extrema* is summarized below.[a-j] See Figures S6-S10 here in the supporting information for the range of sigma hole types (with pictures) for systems considered in this work.

| Group 1[(a)] | Group 2 [(b)] | | Group 13[(c)] | | Group 14 | | Group 15[(g,h)] | | Group 16[(h,i)] | | Group 17[(j)] |
|---|---|---|---|---|---|---|---|---|---|---|---|
| - | R = H | R = F | R = H | R = F | R = H | R = F | R = H | R = F | R = H | R = F | - |
| Li | Be | | B | | C | | N | | O | | F |
| **210.6** | **30.0** | **40.5** | **28.0** | **38.2** | **20.4**[(d)] | 15.2[(e)] | **36.4** | 11.8 | **52.2** | 11.1 | 3.9 |
| Na | Mg | | Al | | Si | | P | | S | | Cl |
| **146.5** | **68.9** | **104** | **65.8** | **84.8** | **30.4**[(e)] | **32.2**[(e)] | **26.3** | **27.6** | 29.8 | 28.0 | 10.3 |
| K | Ca | | Ga | | Ge | | As | | Se | | Br |
| **94.6** | **136** | **136** | **54.0** | **67.8** | **31.9**[(e)] | 33.4[(f)] | **30.7** | **34.5** | **27.6** | **36.8** | 20.6 |
| Rb | Sr | | In | | Sn | | Sb | | Te | | I |
| **79.4** | **135** | **133** | **58.9** | **77.0** | **38.7**[(e)] | **46.9**[(f)] | **37.4** | **42.5** | **36.0** | **45.2** | 32.1 |
| Cs | Ba | | Tl | | Pb | | Bi | | Po | | At |
| **67.8** | **114** | **109** | **51.6** | **62.9** | **39.9**[(e)] | **48.1**[(f)] | **42.9** | **52.7** | **43.7** | **56.4** | **42.2** |

[(a)] •M-I for M = Li, and Na; ○M for M = K, Rb, and Cs. [(b)] Ⓜ for M = Be, Mg, and Ca; •M (in plane) for M = Sr and Ba. [(c)] •M ⊥ for all group 13 molecules. [(d)] C-H•, [(e)] •M-I for all other molecules except the three heaviest $MF_3I$ species. [(f)] For those heavier and more polarizable central atoms (M = Ge, Sn, and Pb), the $V_{s,max}$ for •M-F is quite strong, even stronger in $SnF_3I$ and $PbF_3I$ (in bold in the table) than $V_{s,max}$(I). [(g)] N-H• for $NH_2I$; •N-F for $NF_2I$; •M-I for all other hydrides, and $PF_2I$; •M-F for the three heaviest group 15 fluorides. Only in the case of $NF_2I$ is $V_{s,max}$(I) the most positive extremum. [(h)] We also find very weak positive regions with low $V_{s,max}$ values opposite to lone pair regions in the groups 15 and 16 fluorides and $BiH_2I$. [(i)] M-H• for M = O and S (although $V_{s,max}$(I) is larger for M = S); •M-I for the three heaviest hydrides; •M-F for all fluorides. [(j)] For M = F, Cl, Br, and I, $V_{s,max}$(I) dominates and the next largest $V_{s,max}$ value (shown in this table) is F⊙I. For M = At, the largest $V_{s,max}$ value is I-At•, and it's even larger than $V_{s,max}$(I).

## FIGURES

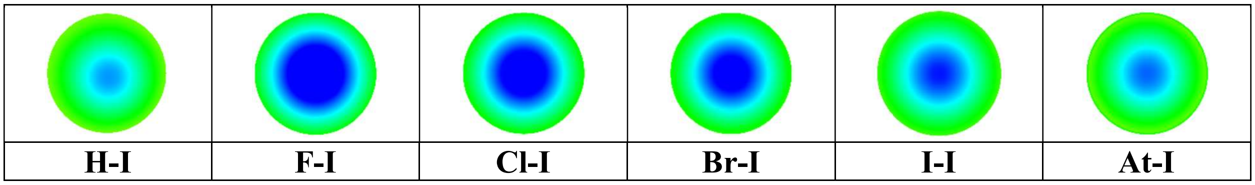


**Figure S1:** The sigma hole on I in the H-I molecule in contrast with sigma holes on I in group 17 iodides. These ESP maps are all plotted in the range ± 5.317 × $10^{-2}$ au, blue (most positive) and red (most negative).

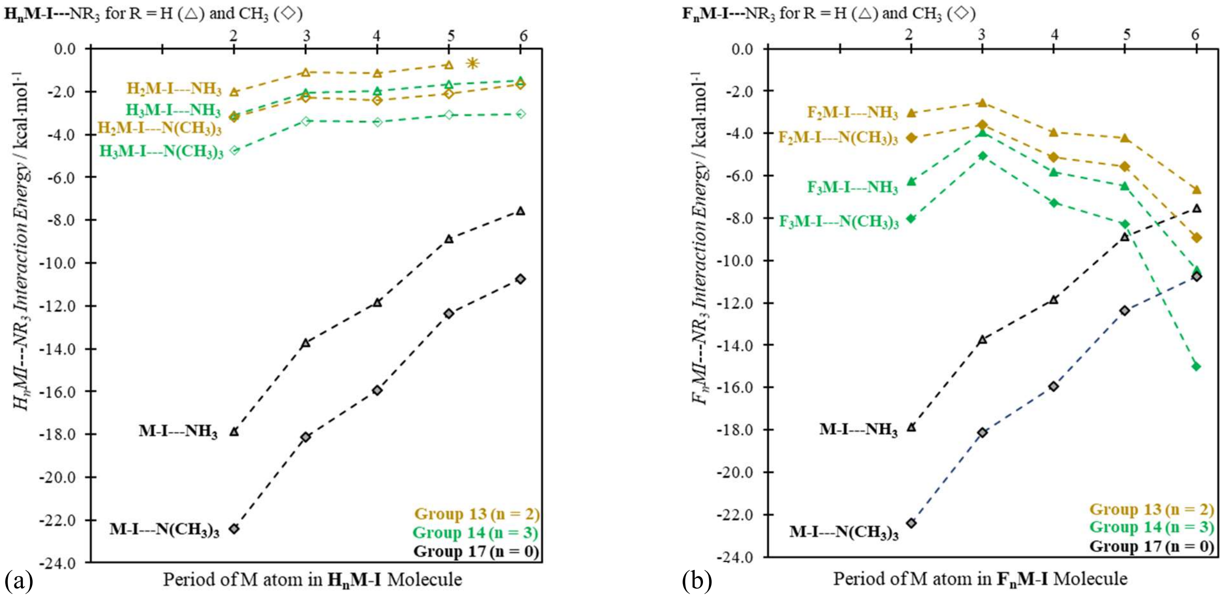


**Figure S2**: Plot of BSSE corrected computed interaction energies, ΔE, in kcal·$mol^{-1}$ units for the $H_nM$-I---Base and $F_nM$-I---Base complexes, where M is a main group atom, and Base = $NH_3$, or $N(CH_3)_3$. * No $H_2Tl$-I---$NH_3$ sigma hole complex was obtained.

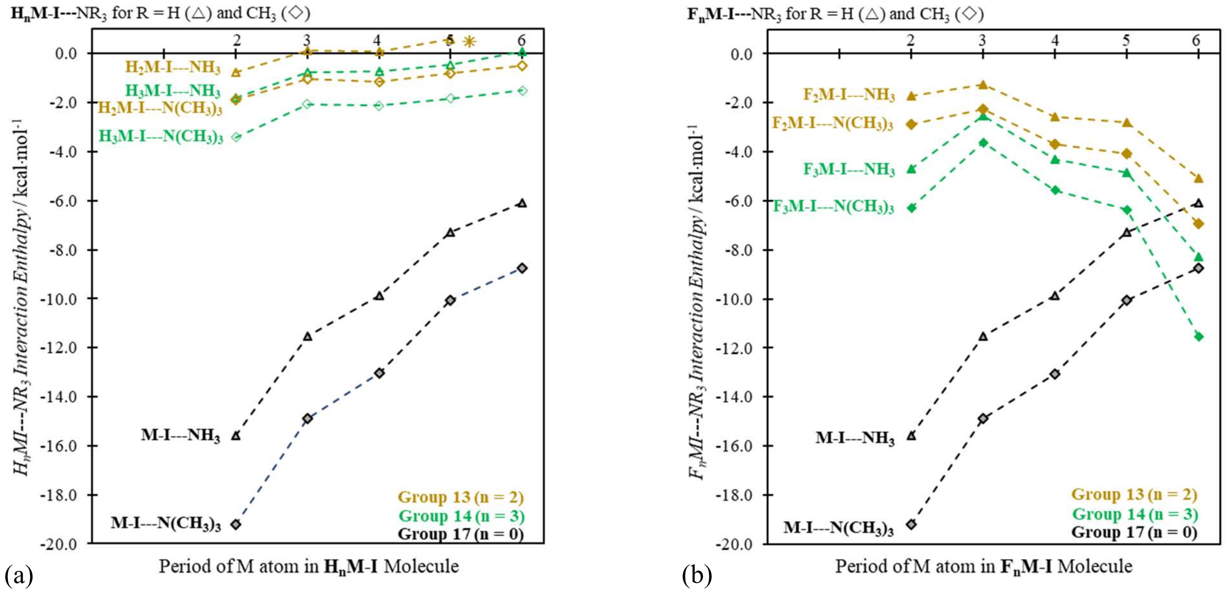


**Figure S3**: Plot of BSSE corrected computed enthalpy changes, ΔH(298.15K), in kcal·$mol^{-1}$ units, for the $H_nM$-I---Base and $F_nM$-I---Base complexes, where M is a main group atom, and Base = $NH_3$, or $N(CH_3)_3$. * No $H_2Tl$-I---$NH_3$ sigma hole complex was obtained.

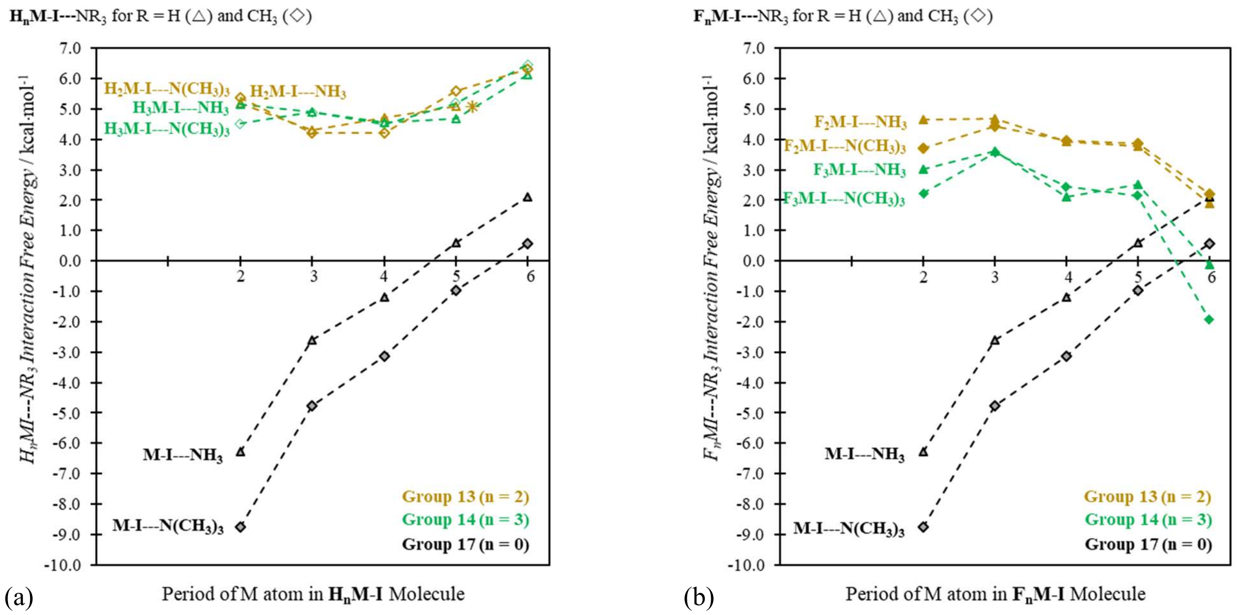


**Figure S4**: Plot of BSSE corrected computed free energy changes, ΔG(298.15K), in kcal·mol$^{-1}$ units, for the $H_nM$-I---Base and $F_nM$-I---Base complexes, where M is a main group atom, and Base = $NH_3$, or $N(CH_3)_3$. * No $H_2Tl$-I---$NH_3$ sigma hole complex was obtained.

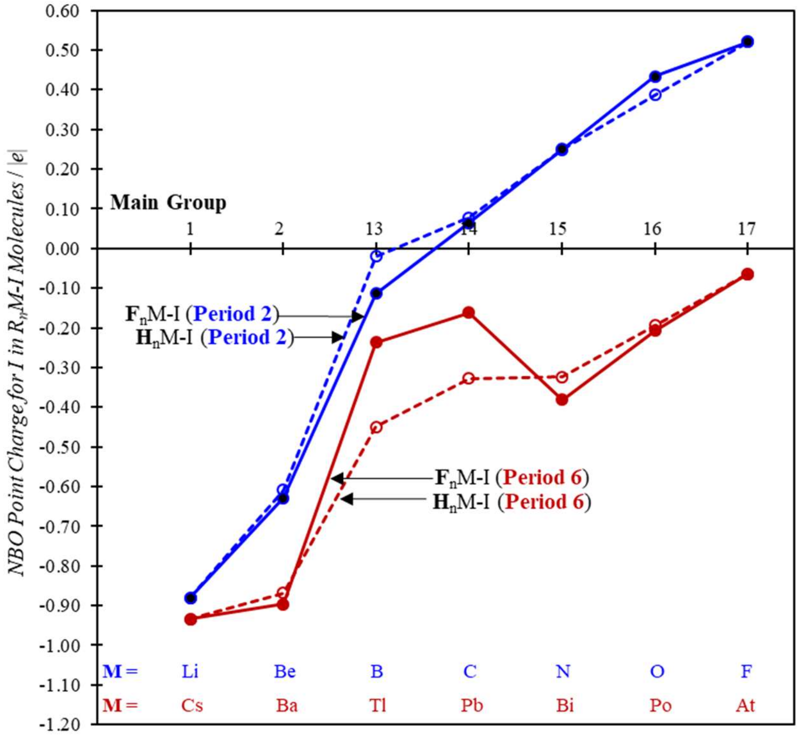


**Figure S5**: Computed Natural Bond Orbital (NBO) point charges for I in $R_nM$-I molecules where M is a period 2 (in blue) or period 6 (in red) main group element and R = H (broken line) and R = F (unbroken line).

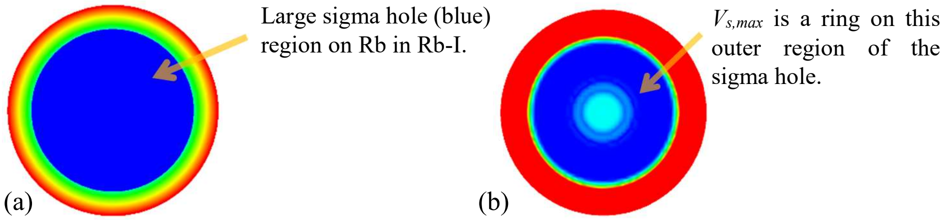


**Figure S6**: [○M; $\boldsymbol{V_{s,max}}$ **(terminal atom) ring**] Map of the electrostatic potentials (ESPs) on the 0.001 isodensity surface plotted (a) in the range ± 5.317 × $10^{-2}$ au, revealing a massive sigma hole, and (b) details of the structure of that sigma hole (on Rb in Rb-I) uncovered using a much narrower range: +0.121 to +0.125 au. Since we use such a narrow range for the color spectrum, the red sections are not necessarily all negative (just < +0.121 au). The surface is plotted with Rb pointing out of the plane of the page and the I atom hidden behind it. The basis for this form of ring extremum remains unclear.

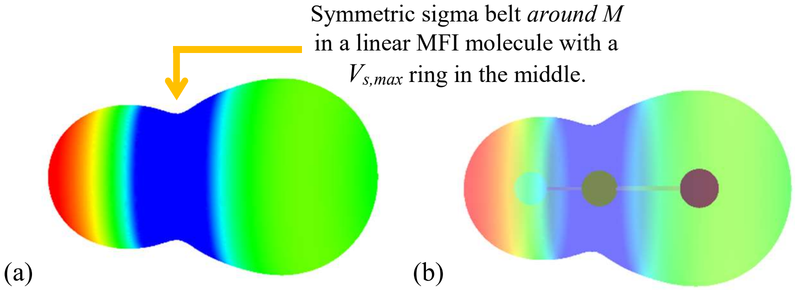


**Figure S7**: [Ⓜ; $\boldsymbol{V_{s,max}}$ **(central atom) ring**] Maps of the electrostatic potentials (ESPs) on the 0.001 au isodensity surface of F-Mg-I, plotted in the range ± 5.317 × $10^{-2}$ au, revealing a massive sigma belt or ring around the central Mg atom. We show (a) an opaque mapped ESP surface, and (b) a semi-transparent form exposing the positions of the F, Mg, and I atomic centers.

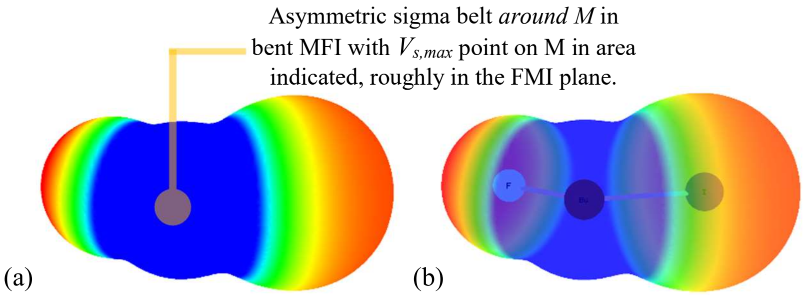


**Figure S8**: [•M (in plane); $\boldsymbol{V_{s,max}}$ **on M in the plane of bent molecule**] Maps of the electrostatic potentials (ESPs) on the 0.001 au isodensity surface of F-Ba-I, plotted in the range ± 5.317 × $10^{-2}$ au, revealing a massive sigma belt or ring around the central Ba atom, compressed on one side due to bending (into the plane of the page). We show (a) an opaque mapped ESP surface, and (b) a semi-transparent form exposing the positions of the F, Ba, and I atomic centers. The bending leads to a local point maximum in the ESP (on M, opposite the bending direction) rather than the ring observed in the linear RMI molecules (Figure S7).

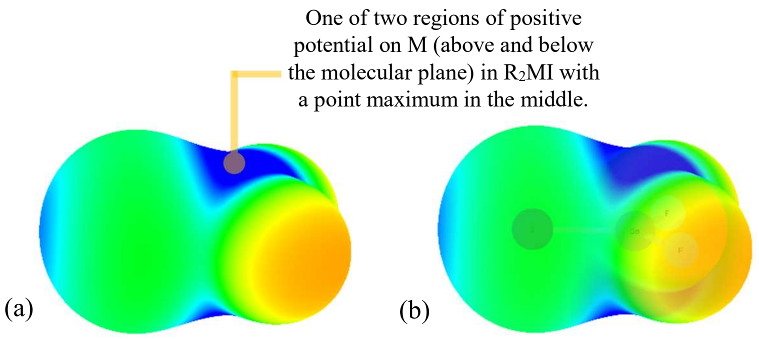


**Figure S9**: [•M ⊥; $V_{s,max}$ **perpendicular to the molecular plane**] Maps of the electrostatic potentials (ESPs) on the 0.001 au isodensity surface of $F_2Ga$-I, plotted in the range ± $5.317 \times 10^{-2}$ au, revealing a significant region of positive potential on the central group 13 (gallium) atom above (shown) and below (partially hidden) the molecular plane. The sigma hole on I is partially visible (on the left of each map). We show (a) an opaque mapped ESP surface, and (b) a semi-transparent form exposing the positions of the atomic centers.

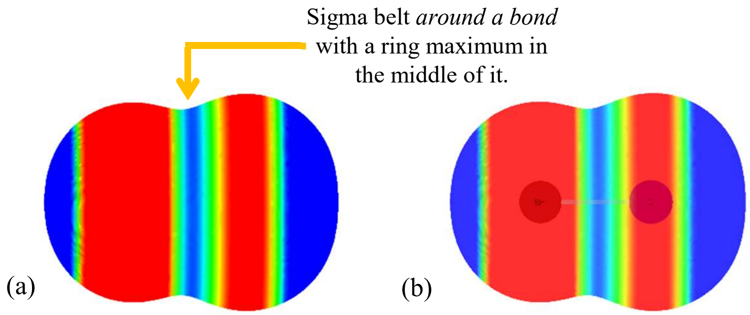


**Figure S10**: [M⊙R; $V_{s,max}$ **(bond) ring**] Maps of the electrostatic potentials (ESPs) on the 0.001 au isodensity surface of Br-I, plotted in the range ± $2.500 \times 10^{-3}$ au, revealing a sigma belt or ring around the Br-I bond. We show (a) an opaque mapped ESP surface, and (b) a semi-transparent form exposing the positions of the Br and I atomic centers.

**References: Sources for Stuttgart-Cologne ECPs and Corresponding Basis Sets**
(For each element, the ECP and Basis Set share the same reference, unless indicated otherwise).

**K, Rb, and Cs**
Ref.: I. S. Lim, P. Schwerdtfeger, B. Metz, H. Stoll, *J. Chem. Phys.* **2005**, *122*, 104103(1-12).

**Sr, and Ba**
Ref.: I. S. Lim, H. Stoll, P. Schwerdtfeger, *J. Chem. Phys.* **2006**, *124*, 034107(1-9).

***ECPs***
**In, Sn, Sb, Pb, and Bi**
Ref.: B. Metz, H. Stoll, M. Dolg, *J. Chem. Phys.* **2000**, *113*, 2563-2569.
**Tl**
Ref.: B. Metz, M. Schweizer, H. Stoll, M. Dolg, W. Liu, *Theor. Chem. Acc.* **2000**, *104*, 22-28.
***Basis Sets.***
**In, Tl, Sn, Pb, Sb, and Bi**
Ref.: K. A. Peterson, *J. Chem. Phys.* **2003**, *119*, 11099-11112.

**Te, Po, and At**
Ref.: K. A. Peterson, D. Figgen, E. Goll, H. Stoll, M. Dolg, *J. Chem. Phys.* **2003**, *119*, 11113-11123.

**I**
Ref.: K. A. Peterson, B. C. Shepler, D. Figgen, H. Stoll, *J. Phys. Chem. A* **2006**, *110*, 13877-13883.

**Full Gaussian 16 Reference**

Gaussian 16, Revision B.01, Frisch, M. J.; Trucks, G. W.; Schlegel, H. B.; Scuseria, G. E.; Robb, M. A.; Cheeseman, J. R.; Scalmani, G.; Barone, V.; Petersson, G. A.; Nakatsuji, H.; Li, X.; Caricato, M.; Marenich, A. V.; Bloino, J.; Janesko, B. G.; Gomperts, R.; Mennucci, B.; Hratchian, H. P.; Ortiz, J. V.; Izmaylov, A. F.; Sonnenberg, J. L.; Williams-Young, D.; Ding, F.; Lipparini, F.; Egidi, F.; Goings, J.; Peng, B.; Petrone, A.; Henderson, T.; Ranasinghe, D.; Zakrzewski, V. G.; Gao, J.; Rega, N.; Zheng, G.; Liang, W.; Hada, M.; Ehara, M.; Toyota, K.; Fukuda, R.; Hasegawa, J.; Ishida, M.; Nakajima, T.; Honda, Y.; Kitao, O.; Nakai, H.; Vreven, T.; Throssell, K.; Montgomery, J. A., Jr.; Peralta, J. E.; Ogliaro, F.; Bearpark, M. J.; Heyd, J. J.; Brothers, E. N.; Kudin, K. N.; Staroverov, V. N.; Keith, T. A.; Kobayashi, R.; Normand, J.; Raghavachari, K.; Rendell, A. P.; Burant, J. C.; Iyengar, S. S.; Tomasi, J.; Cossi, M.; Millam, J. M.; Klene, M.; Adamo, C.; Cammi, R.; Ochterski, J. W.; Martin, R. L.; Morokuma, K.; Farkas, O.; Foresman, J. B.; Fox, D. J. Gaussian, Inc., Wallingford CT, 2016.
